# Spinal Cord Injury Induces Permanent Reprogramming of Microglia into a Disease-Associated State Which Contributes to Functional Recovery

**DOI:** 10.1523/JNEUROSCI.0860-21.2021

**Published:** 2021-10-06

**Authors:** Ramil Hakim, Vasilios Zachariadis, Sreenivasa Raghavan Sankavaram, Jinming Han, Robert A. Harris, Lou Brundin, Martin Enge, Mikael Svensson

**Affiliations:** ^1^Department of Clinical Neuroscience, Karolinska Institutet, Stockholm S-17177, Sweden; ^2^Center for Molecular Medicine, Karolinska Hospital at Solna, Stockholm S-17176, Sweden; ^3^Department of Oncology-Pathology, Karolinska Institutet, Stockholm S-17176, Sweden

**Keywords:** Basso mouse scale, disease-associated microglia, scRNAseq, spinal cord injury

## Abstract

Microglia are resident myeloid cells of the CNS. Recently, single-cell RNA sequencing (scRNAseq) has enabled description of a disease-associated microglia (DAM) with a role in neurodegeneration and demyelination. In this study, we use scRNAseq to investigate the temporal dynamics of immune cells harvested from the epicenter of traumatic spinal cord injury (SCI) induced in female mice. We find that as a consequence of SCI, baseline microglia undergo permanent transcriptional reprogramming into a previously uncharacterized subtype of microglia with striking similarities to previously reported DAM as well as a distinct microglial state found during development. Using a microglia depletion model we showed that DAM in SCI are derived from baseline microglia and strongly enhance recovery of hindlimb locomotor function following injury.

**SIGNIFICANCE STATEMENT** Although disease-associated microglia (DAM) have been the subject of strong research interest during recent years (Keren-Shaul, 2017; Jordão, 2019), their cellular origin and their role in “normal” acute injury processes is not well understood. Our work directly addresses the origin and the role of DAM in traumatic injury response. Further, we use a microglia depletion model to prove that DAM in spinal cord injury (SCI) are indeed derived from homeostatic microglia, and that they strongly enhance recovery. Thus, in this work we significantly expand the knowledge of immune response to traumatic injury, demonstrate the applicability to human injury via our unique access to injured human spinal cord tissue, and provide the community with a comprehensive dataset for further exploration.

## Introduction

Microglia are cells with mesodermal origin that reside in evenly distributed individual compartments throughout the CNS. The baseline population of microglia is stable (Ajami et al., 2007; Mildner et al., 2007), but is activated into a proliferative and mobile state on tissue injury (Sievers et al., 1994; Eder et al., 1998; Schilling et al., 2001; Hanisch and Kettenmann, 2007; Davoust et al., 2008; Colton and Wilcock, 2010; Kettenmann et al., 2011). Activated microglia can proliferate and migrate to the site of injury where they phagocytose debris and cells (Kettenmann et al., 2011), which has been thought to aid in tissue healing. More recently, studies using single-cell RNA sequencing (scRNAseq) have identified alternate microglial cell states during disease. Such disease-associated microglia (DAM) have been observed during several processes including aging, Alzheimer's disease (AD; Keren-Shaul et al., 2017; Mathys et al., 2017; Friedman et al., 2018; Mrdjen et al., 2018; Olah et al., 2018; Tay et al., 2018; Hammond et al., 2019), multiple sclerosis (Jordão et al., 2019), neurodegeneration (Tay et al., 2018), inflammation (Sousa et al., 2018), demyelination (Masuda et al., 2019), and in response to neuronal apoptosis (Krasemann et al., 2017), raising questions concerning the role and function of DAM.

Although DAM have been the subject of strong research interest during recent years, their cellular origin and their role in acute injury processes is not well understood. In this study, we investigated the response of healthy tissue to injury, in the context of traumatic spinal cord injury (SCI). To obtain a detailed view of the temporal dynamics of immune cells after SCI, we applied scRNAseq to analyze 3687 single cells taken from healthy tissue and from injured spinal cord at a wide range of time points ranging from 0.5 h to 90 d post-SCI. Thus, we were able to trace an unbroken path between the acute and chronic tissue state of the injury. We determined that following SCI baseline microglia undergo permanent transcriptional reprogramming, resulting in a subtype of microglia with striking similarities to DAM, cells which we could also observe in human SCI. Using a microglial depletion model we showed that DAM in SCI are derived from baseline microglia and do in fact enhance recovery of hindlimb locomotor function in mice subjected to SCI.

## Materials and Methods

### Experimental design

#### Main experiment

C57BL/6J mice were subjected to severe contusion SCI. At 0.5 h, 2 h, 6 h, 24 h, 36 h, 3 d, 7 d, 21 d, and 90 d post-SCI the injury epicenters were harvested. Healthy tissue was included as reference. Following tissue dissociation all CD45^+^ cells were isolated and sequenced individually. Three to five animals were used per time point. Cell suspensions from the individual animals were pooled into one sample for each time point.SCI epicenter tissue sections were stained for APOE, CST7, IGF1, IBA1, and TMEM119 at each time point. Healthy tissue was included as reference. Three to five animals were investigated for each time point and stain. Proliferation of CD45^+^ cells was investigated at 7 and 21 d post-SCI cells using flow cytometry. Three animals were investigated at each time point.

#### Origin of DAM

Cx3cr1^+^ cells were depleted by administration of tamoxifen to C57BL/6J-CX3CR1^CreER/+^Rosa26^DTA/+^ mice at 9, 8, and 7 d prior to SCI. Severe contusion SCI was induced and CD45^+^ cells were isolated and sequenced individually at 7 and 21 d post-SCI. Hind limb function was assessed during 90 d post-SCI. Wild-type animals were used as control. Each experimental group contained three or four animals.

#### Human tissue

Sections of injured human spinal cord were stained for APOE, CST7, IGF1, IBA1.

#### Bulk sequencing of spinal cord

CX3CR1+CD19-LY6G-CD3-NK1.1-SIGLEC-F- cells were isolated and subjected to sequencing in bulk at 7 and 21 d post-SCI. Healthy tissue was used as reference.

### Mice

Wild-type female C57BL/6J mice (Scanbur), CX3CR1-GFP (005582, The Jackson Laboratory) and CX3CR1^CreER/+^Rosa26^DTA/+^ (in-house breeding of 021160 and 009669 from The Jackson Laboratory by the R.A.H. lab) were used. Animal care and experiments were conducted in line with ethical permits (N196/15, N12317-2017, N138/14) approved by the local ethical committee (Stockholm, Sweden) and in line with local guidelines at Karolinska Institutet. Adult (10–15 weeks) females were used in all experiments.

### Genotyping

#### Cx3cr1-gfp

Protocols from The Jackson Laboratory were implemented. Briefly, ear biopsies were incubated for 1 h at 95°C in 100 µl solution [1:200 5 m NaOH (in-house), 1:2500 0.5 m EDTA (Invitrogen, 15575-038) in Milli-Q]. Samples were placed on ice and 100-µl solution (1:25 Tris-HCl; Merck Millipore, T5941) was added. 4 µl of the suspension was added to 16 µl of master mix (62.5% REDExtract-N-Amp PCR (Sigma, R4775), 28% nuclease free-water, 3% primer mutant, 3% primer wild type and 3% primer common). Working stock of primers (Eurofins Genomics; Table 1) was added at 20 μm. Suspension was added to a PCR plate (Microamp Optical 96-well Reaction Plate, Life Technologies N8010560). After centrifugation at 200 × *g* for 60 s, the PCR plate was loaded onto a PCR instrument. A thermal cycling protocol was initiated with polymerase activation and denaturation at 94°C for 2 min, followed by 10 cycles of denaturation at 94°C for 20 s and annealing/extension at 65°C for 15 s (decreased 0.5°C/cycle) and 68°C for 10 s. This was followed by 28 cycles of denaturation at 94°C for 15 s, annealing/extension at 60°C for 15 s and 72°C for 10 s followed by 2 min at 72°C. Following thermal cycling the samples were added into an agarose gel (E-Gel Agarose Gel with SYBR Safe DNA Gel stain 2%, Invitrogen G521802) in combination with 20 µl DNA ladder (GeneRuler 100 bp, Invitrogen SM0241) and connected to a power-supply (Bio-Rad, Power-Pac 100) by which 90 V was delivered for 15–20 min. Heterozygotes were identified as expressing fluorescence at 337 and 485 bp. Mutants (485 bp only) and wild type (337 bp only) were killed.

**Table 1.** Primer sequences for genotyping

Strain	Primer	Sequence 5′->3′
C57BL/6J-Cx3cr1^GFP^	moIMR0003	GGG CCA GCT CAT TCC TCC CAC TCA T
	oIMR4281	CCA CAG GAT TTC AGC CTG AAC TTT G
	oIMR4282	CGT GCA CTA TGC TCA GAT ATC TGT C
Cx3cr1^C^*^re^*^ER+^Rosa26^DTA+^	Rosa26 mutant forward	CGA CCT GCA GGT CCT CG
	Rosa26 mutant reverse	CTC GAG TTT GTC CAA TTA TGT CAC
	Rosa26 WT forward	CGT GAT CTG CAA CTC CAG TC
	Rosa26 WT reverse	GGA GCG GGA GAA ATG GAT ATG

GFP: Green Fluorescent Protein; DTA: Diphtheria Toxin A; WT: Wild-Type.

#### Cx3cr1^creer/+^Rosa26^DTA/+^

DNA was prepared from ear biopsies. Using specific primers (Table 1) and PCR amplification, the genotype for each mouse was determined. The PCR reaction was conducted using a thermal cycler (Applied Biosystems, 2720) at 94°C for 5 min; 35 cycles at 94°C for 30 s, 60°C for 60 s, and 72°C for 60 s followed by 72°C for 2 min. The DNA fragments were measured on an agarose gel (4%) using a GelRed fluorescent nucleic acid gel stain.

### Induction of SCI in mice

Intraperitoneal injection anesthesia consisting of 0.5 mg/kg medetomidin intraperitoneal (Domitor vet., Orion Pharma Animal Health 1 mg/ml) and 75 mg/kg ketamine intraperitoneal (Ketador vet., Salfarm Scandinavia, 100 mg/ml) was administrated. Analgesics were administrated subcutaneously at induction using 0.05 mg/kg buprenorfin subcutaneously (temgesic, 0.3 mg/ml) and 5 mg/kg karprofen subcutaneously (Rimadyl, 50 mg/ml). The skin above the thoracic vertebrae was incised, the paravertebral muscles separated and the vertebral column stabilized using bilateral fixators in a stereotaxic frame (Model 900 & 900-c, Kopf). The dorsal part of the vertebrae was removed using a surgical drill (Anspach, EMAX 2). A 75 kdyn contusion SCI was induced at Th10 (Infinite Horizon, IH-0400). Bupivakain (Marcain, Aspen Nordic 2.5 mg/ml) was injected into the muscles surrounding the injury and the skin was sutured (Ethicon, Vicryl 4.0). Post-operative analgesic treatment and care was given twice daily for a total of 3 d after surgery. Bladders were compressed manually until reflexive bladder emptying returned. The humane endpoint was set at 25% weight loss and was evaluated weekly.

### Harvesting mouse spinal cords

Animals were euthanized using a lethal dose of pentobarbitalnatrium (Apotek Produktion & Laboratorier, 60 mg/ml) and transcardially perfused with ice-cold 1× DPBS using a peristaltic pump (Watson Marlow, 120S). Animals for histologic evaluation were further perfused with 4% paraformaldehyde (PFA; Thermo Fisher Scientific 28908) and postfixed in 4% PFA at 4°C O/N. Tissue for single-cell analysis was harvested from the SCI epicenter, defined as the site of impact ±3 mm along the cranial-caudal axis in the mouse. Even in our highly controlled setting, the composition of the injury (size of cystic cavity, size of scar, degree of hematoma, force transmission and injury severity) varied slightly between the animals. Thus, a pool of three to five mice were used for each time point. This strategy allowed us to capture the heterogeneity in the immune cell response more accurately at the different time points and at the same time being able to pursue a cost-efficient and time-efficient approach.

### Harvesting human spinal cords

Human SCI samples were retrieved from an ongoing clinical trial [Safety and Efficacy of SC0806 (fibroblast growth factor 1 and a device) in traumatic SCI subjects] approved by the Swedish Medical Products Agency Study Protocol SC0806-A101. The study is currently ongoing at Karolinska University Hospital (Stockholm, Sweden) using ethical permits 2013/2257-31/1, 2015/1436-31, and 2019-03671. All research subjects gave their signed written informed consent. Briefly, the injured segment of the spinal cord was surgically removed in patients with SCI in the chronic stage. The rostral 1/3 of the dissected tissue was used for histologic evaluation of immune cells.

### Dissociation of spinal cords

The SCI epicenters from three to five spinal cords/mice from the same treatment groups were pooled. This approach was implemented to limit variability within each time point. Spinal cords were dissociated using enzymatic dissociation with Papain (10 U/ml; Worthington, L5003126) in L-15 medium (Thermo Fisher Scientific, 11415064). Single-stranded and double-stranded DNA were digested using 200 U/ml DNase (Sigma, D7291). Papain was inactivated using 1% BSA (Invitrogen, 15 260–037) followed by myelin removal using 30% Percoll gradient (Sigma, P1644) at 750 × *g* for 10 min at 4°C with slow brake. Cells were resuspended in fluorescence-activated cell sorting (FACS) buffer (1% BSA, 2 mm EDTA, 25 mm HEPES; Sigma, H0887) and passed through 100-µm (Corning, 431752) and 40-µm (Corning, 431750) cell strainers. Papain dissociation was used in favor of approaches such as mechanical dissociation and/or collagenase since we observed a superior cell survival when using Papain. The extent to which Papain affects the transcriptome of the cells is difficult to assess but Papain was used for all treatment groups. Dead cells and debris were removed using MACS Dead Cell Removal kit (Miltenyi Biotec, 130-090-101) using manufacturer's protocol. Cells were collected and resuspended in 100-µl FACS buffer and incubated in Fc-lock (CD16/32; BD, 553141) 1 µg/10^6^ cells for 5 min on ice. Preconjugated antibodies (Table 2) were added onto the cell suspension at a concentration of 1 µg/10^6^ cells and the cells were stained for 30 min on ice while protected from light.

**Table 2.** Antibodies for FACS and histology

Type	Host	Target	Conjugation	Manufacturer	ID
Primary	Rabbit	TMEM119	NA	Abcam	ab209064
	Goat	IBA1	NA	Abcam	ab48004
	Rabbit	IBA1	NA	Wako	019–19741
	Mouse	APOE	NA	Abcam	ab1907
	Rabbit	IGF1	NA	Abcam	ab40657
	Rabbit	CST7	NA	Nordic Biosite	12073–1-AP
Secondary	Donkey	Rabbit	Alexa Fluor 488	Abcam	ab150073
	Donkey	Mouse	Alexa Fluor 488	Abcam	ab150105
	Donkey	Rabbit	Alexa Fluor 594	Abcam	ab150076
	Donkey	Mouse	Alexa Fluor 594	Abcam	ab150108
	Donkey	Goat	Alexa Fluor 594	Abcam	ab150132
	Donkey	Rabbit	Alexa Fluor 647	Abcam	ab150075
	Donkey	Goat	Alexa Fluor 647	Abcam	ab150135
Preconjugated	Rat	CD11B	PerCP-Cy5.5	Biolegend	101227
	Rat	CD11B	APC	BD Biosciences	553312
	Rat	CD11B	BV421	BD Biosciences	562605
	Rat	CD45	PE/Cy7	Biolegend	103113
	Rat	CD45	PE	BD Biosciences	553081
	Rat	LY6C	PE	Biolegend	128007
	Rat	LY6G	BV421	BD Biosciences	562737
	Rat	LY6G	V450	BD Biosciences	560603
	Rat	CD19	BV421	BD Biosciences	562701
	Rat	SIGLEC-F	BV421	BD Biosciences	562681
	Hamster	CD3E	BV421	BD Biosciences	562600
	Mouse	NK1.1	BV421	Biolegend	108741
	Rat	CD14	FITC	Biolegend	123307
	Mouse	CD64	PerCP-Cy5.5	Biolegend	139308
Live/dead	NA	Free amines	Near-IR	Thermo Fisher	L34959
Counterstain	NA	dsDNA	Hoechst 33258	Invitrogen	H3569
Fc block	Rat	CD16/32	NA	BD Biosciences	553141

NA: Not Applicable.

### Sorting single cells from spinal cord

Following staining the cells were washed in FACS buffer and eventually resuspended in 500 µl FACS buffer; 0.5-µl live/dead dye (Thermo Fisher Scientific, L34959) was added to the suspension. FACS was conducted using a Sony SH800S cell sorter. A mixture of unstained cells from healthy and injured spinal cord in equal volumes was used as negative control when setting compensations. CD45^+^ cells from the spinal cords were sorted into Hard-Shell 384-well PCR plates (Bio-Rad, hsp3805) containing 2-µl lysis buffer in each well (Tables 3, 4). Plates were covered with a seal (Bio-Rad, MSF1001), centrifuged at 4000 rpm (Hettich, Universal 320R) for 2 min, then snap-frozen on dry-ice and stored at −80°C.

**Table 3.** SmartSeq2, library preparation

Type	Ingredient	Ratio	Manufacturer	Identifier
Lysis buffer	H_2_O	1:53	NA	
	Recombinant RNase inhibitor	1:40	Takara	2313A
	ERCC 1:600,000	1:40	In-house	
	10% Triton X-100	1:50	Sigma	93443
	10 mm dNTP	1:4	In-house	
	100 µm dT	1:40	In-house	
Reverse transcription mix	SMARTScribe	1:6.32	Takara	639538
	Recombinant RNase inhibitor	1:24	Takara	2313A
	5× first strand buffer	1:3	Takara	639538
	100 mm DTT	1:12	Promega	P117A
	5 m betaine	1:3	Sigma	B0300
	1 m MgCl_2_	1:100	Sigma	M1028
	100 µm TSO	1:60	In-house	
	H_2_O	1:43	NA	
Preamplification mix	H_2_O	1:7	NA	
	Kapa HiFi HotStart ReadyMix (2×)	1:1.2	Kapa Biosystems	KK2601
	10 µm ISPCR primer	1:60	In-house	
	λ Exonuclease	1:133	New England Biolabs	M0262S
Tn5 mix	H_2_O	1:1.7	NA	
	TAPS-PEG	1:3.6	In-house	
	Tn5 stock	1.7.2	In-house	
PCR/bar code mix	H_2_O	1:1.47	NA	
	5× buffer	1:3.9	KAPA	KB2500
	dNTPs	1:26	KAPA	KN1009
	Hifi	1:39	KAPA	KE2004

NA: Not Applicable.

**Table 4.** OligodT, TSO, and primer sequences

Type	Sequence	Source
OligodT	5′-AAGCAGTGGTATCAACGCAGAGTACT30VN-3′	Picelli et al. (2014)
TSO	5′-AAGCAGTGGTATCAACGCAGAGTACATrGrG+G-3′	
ISPCR	5′-AAGCAGTGGTATCAACGCAGAGT-3′	

TSO: Template-Switching Oligonucleotide; ISPCR: in situ PCR.

### Library preparation and sequencing of single cells

Full-leng cDNA and sequencing libraries were prepared using the Smart-Seq2 protocol (Picelli et al., 2014). Briefly, primers were annealed at 72°C for 3 min (Bio-Rad, 100°C Touch Thermal Cycler) followed by immediate reverse transcription and template switching by adding 3-µl (Eppendorf, epMotion 5073) reverse transcription mix (Tables 3, 4) to each well and kept at 42°C for 90 min and 70°C for 5 min. DNA was preamplified by adding 7.5 µl of preamplification mix (Tables 3, 4) into each well and kept at 37°C for 30 min, 95°C for 3 min, 22 cycles of: 98°C for 20 s; 67°C for 15 s; 72°C for 4 min followed by 72°C for 5 min. DNA was purified by adding solid phase reversible immobilization (SPRI) beads at a ratio of 0.75:1, incubated for 5 min at room temperature (RT), washed twice with 30 µl 80% EtOH (Solveco, 1000), air dried for 15 min and eluted in 15 µl EB buffer (QIAGEN, 19086). A magnetic plate (custom made) was used for separation of the beads during processing. DNA concentration was measured in 16 random wells from each 384-well plate using a step-wise pattern with QUBIT dsDNA HS Assay kit (Q33231); 3-µl sample was added to 97-µl buffer in a 96-well plate (Invitrogen, M33089) and concentrations were measured and compared with standard (Tecan spark 10 M, ex/em 485/528 nm). DNA was then diluted to a concentration of 0.15 µg/µl using RNase-free H_2_O. Tagmentation was performed by adding 1.8 µl Tn5 mix to each well in a 384-well plate (Table 3); 0.7 µl cDNA was then added into each well containing the Tn5 mix. The plate was vortexed (Thermo Fisher Scientific, 88880017TS), centrifuged and kept at 55°C for 10 min. Tn5 was inactivated by adding 1 µl 0.1% SDS (Sigma, 71725) to each well. Plate was vortexed and incubated at RT for 7 min. Libraries were barcoded by adding 19.5 µl PCR/bar code mix into each well now containing 3.5-µl sample (Table 3). A 2-µl primer (3.75 μm/primer) was finally added to each well. PCR/barcoding was then conducted at 72°C for 3 min, 95°C for 3 min followed by 12 cycles of: 95°C for 15 s; 55°C for 30 s; 72°C for 45 s. DNA was pooled (5 µl from each well) into an Eppendorf tube. Clean-up, as described above, was repeated and DNA was eventually eluted in 30-µl EB buffer. Concentration was again measured using QUBIT (3.0 Fluorometer) before 37 bp paired-end sequencing with dual index 8 bp using 2.6 pm on a 309 bp peak on Illumina NextSeq.

### scRNAseq data analysis

#### Demultiplexing and alignment

Demultiplexed FASTQ files were trimmed, adaptor sequences removed and aligned to the Genome Reference Consortium Mouse Build 38 (GRCm38, build mm10) using STAR (Dobin et al., 2013). Following removal of duplicate reads HT-seq was used for obtaining raw transcript counts. Eventually, the count matrix was exported into R (statistical computing software, v3.6.0; Team} RC, 2016) for downstream analysis (Table 5).

**Table 5.** Tools for data manipulation

Tool	Version	Author	URL
STAR RNAseq aligner	2.5.2	Dobin et al. (2013)	https://github.com/alexdobin/STAR
Subread/feature counts	1.5.1	Liao et al. (2014)	http://subread.sourceforge.net/
HTSeq	0.11.2	Anders et al. (2014)	https://github.com/simon-anders/htseq
R	3.6.0	R Core Team	https://www.R-project.org/
Seurat	3.0.0	Butler et al. (2018)	https://github.com/satijalab

#### Data preprocessing and quality control

Quality control wells (bulk and empty wells), ERCC, non-genes and genes present in zero genes were removed. Normalized counts per million (CPM) was estimated for each gene and cell by dividing the counts for each gene and cell with the sum of all counts for that particular cell, multiplying by 10^6^ and adding 1 (pseudocount) before log2-transformation. Cells with *Actb* expression in the first percentile were removed (Extended Data Fig. 1-1*A*). Log2 of features was plotted against log2 of counts to determine the cutoff for cells with low number of counts and features. The minimum counts per cell was set at 2^16^ (Extended Data Fig. 1-1*B*). The filtered data had a median sequencing depth of 606,166 reads/cell (IQR: 643,913) and 2191 genes/cell (IQR: 1228). Out of 4734 sequenced cells 3687 cells and 20,469 genes with a mean of 307 cells/condition remained for downstream analysis. No significant batch effects between samples sorted on different occasions were observed.

#### Clustering and dimensionality reduction

R (Team} RC, 2016) package Seurat (v3.0.0) was implemented for downstream analysis of the preprocessed data. Normalization was conducted with scale.factor = 10^4^ using NormalizeData(). Highly variable genes were defined as having a mean log2(cpm)>0.3 (mean.Cutoff) and normalized dispersion (dispersion.Cutoff)>0.5 keeping other parameters in FindVariableFeatures() at default (Extended Data Fig. 1-1*C*). Data were scaled using ScaleData() while RunPCA() was used for determining and detecting the first 50 principal components (PCs). The standard deviation for the 50 PCs were plotted to determine the highly variable PCs (Extended Data Fig. 1-1*D*). The first 17 PCs were selected for construction of the shared nearest neighbor (SNN) graph using FindNeighbors(). The SNN graph was used for identification of clusters using FindClusters() implementing the Louvain algorithm using resolution = 1.3. Resolution was set at 1.3 following evaluation of clustering using resolutions between 0.5 and 4. Hallmark genes for immune cells were used for evaluation of the clusters. A proper resolution was deemed to be one which did not overcluster or undercluster the data. Dimensionality reduction was conducted using RunTSNE(). The first 15 dimensions were estimated with seed.use = 132 and perplexity = 70. When including immune cells sorted following microglial depletion (plate IDs VZC01001, VZC01101) the key settings mentioned above were adjusted to: FindNeighbors (dims = 1:20), FindClusters (resolution = 0.75), RunTSNE (dims = 1:16, seed.use = 246), while the other settings were as above.

#### Pseudotime ordering of microglial cells

Pseudotime analysis was only conducted for microglial cells (*bMicroglia*, *aMicroglia*, *pmMicroglia*, *maMicroglia*, *DAM*). Centroids (mean tSNE-1 and mean tSNE-2) were estimated for each condition. The Euclidian distance was estimated from each cell to each centroid, each cell being assigned to the closest centroid. Cells were ordered along pseudotime by estimating a principal curve using the centroids as anchors. A linear model was estimated for all ∼20,000 genes. The 10 genes with the most positive slopes and the 10 genes with the most negative slopes were included in A heat map. The genes were ordered vertically using hierarchical clustering. log2(cpm) expression was reported for each microglial cell over pseudotime for a selected set of genes.

#### Cluster marker genes and differential gene expression analysis

Marker genes for each cluster were identified using *FindAllMarkers()* using logfc.threshold=log(2) and other settings at default. Differentially expressed genes between selected clusters were identified using the edgeR (Robinson et al., 2010) and limma (Ritchie et al., 2015) packages in R (Team} RC, 2016) using the trimmed mean of M values (TMM) method for calculation of normalization.

#### Integration with peer datasets

The Seurat R package (v3.0.0) was implemented for integrating the current data with the three published datasets (Table 6). Each dataset was normalized using NormalizeData() and variable features were identified using FindVariableFeatures() using selection.method=vst and nfeatures = 2000. Anchors were identified for the joint dataset using FindIntegrationAnchors() and the data were integrated using IntegrateData() both functions using the 30 first dimensions. Data were scaled using ScaleData() and RunPCA() was used to determine and detect the first 50 PCs with seed.use = 42. The first 10 PCs were selected for construction of the SNN graph using FindNeighbors(). The SNN graph was used for identification of clusters using FindClusters() implementing the Louvain algorithm using resolution = 1. Dimensionality reduction was conducted using RunTSNE() using the first 10 dimensions with seed.use = 246 and perplexity = 70. In the first part of the analysis, the current data were integrated with data published by Li et al. (2019), while all four datasets, adding data from Masuda et al. (2019) and Mathys et al. (2017), were integrated at once in the end. The same settings were used during both integrations except in FindNeighbors() which was set to use the first 13, rather than 10, dimensions during the integration including four datasets.

**Table 6.** Datasets

	GEO accession	PMID
This study	GSE142849	-
Li et al. (2019)	GSE123022, GSE123024	30606613
Masuda et al. (2019)	GSE120744, GSE120745	30760929
Mathys et al. (2017)	GSE103334	29020624

GEO: Gene Expression Omnibus; PMID: PubMed ID.

#### Correlogram of transcription factors

Transcription factors defining each microglial cell cluster were identified using *FindAllMarkers()* using a minimum of 10% difference in fraction between clusters (min.diff.pct), logFC threshold at 0.4 (logfc.threshold) and genes that were detected in at least 30% of the cells in each cluster (min.pct), respectively. Top transcription factors for each cluster were selected based on logFC [fulfilling false discovery rate (FDR) < 0.05], and a correlogram was constructed indicating the correlation and cell type for each transcription factor.

#### Index sorting and FACS trace-back

Surface expression data were exported from the FACS apparatus. Because of slight variations in voltage settings between sorting sessions the expression data were separately normalized for each marker and plate. This allowed us to plot the expression for all cells at once in the same tSNE. FACS trace-back is defined as plotting all sorted and sequenced cells in a reconstructed FACS-plot (CD45 vs BSC-A) containing all analyzed cells to indicate granularity and CD45 expression of different cell types.

### Immunohistochemistry and confocal microscopy

Following postfixation and cryo-protection in sucrose (Sigma, S9378) the spinal cords were mounted using 40% sucrose in 1× PBS; 30-µm free-floating transversal sections were produced (Leica, SM2000R) and kept in 1× PBS with 0.1% NaN_3_ (Sigma, S-2002) in a 24-well plate (Nunc, 142475). Primary antibody (Table 2) was diluted in blocking buffer: 0.3% Triton X-100 (Sigma, 93443), 5% normal donkey serum (Sigma, S30), 5% BSA (Sigma, A2153), 1× PBS, and NaN_3_. Sections were incubated in primary antibody with gentle shaking (IKA, MS 3 digital) at 4°C O/N. Sections were washed three times in 1× PBS with gentle shaking and incubated in secondary antibody (Table 2) at 4°C O/N. Sections were washed three times in 1× PBS and then incubated in nucleic acid stain (Hoechst 33258, Invitrogen H3569) for 20 min at 4°C with gentle shaking. Slides were once again washed three times and placed on slides (VWR, Superfrost Plus, 48311-703). A small amount of Mowiol (Sigma, 81381) was added and the sections protected using a cover slip (Marienfeld, 010243). The stained sections were imaged using a confocal microscope (Zeiss, LSM 880 Airyscan).

### Immunocytochemistry

Sorted microglia/macrophages were plated at a density of 10^3^ cells/cm^2^ on glass slides (Nunc Lab-Tek Chamber Slide, 177402). Cells were subcultured in culture medium: 90% DMEM/F12 (Invitrogen, 11320033), 10% fetal calf serum (Sigma, F2442), M-CSF 10–20 ng/ml, and 1% Pen-Strep (Invitrogen, 15140122). Four days after plating, the cells were fixed using 2% PFA in 1× PBS for 30 min. Fixed cells were incubated with primary antibody (Table 2) diluted in blocking buffer at 4°C O/N. Following wash three times in 1× PBS the cells were incubated with secondary antibody (Table 2) diluted in 1× PBS for 1 h at RT. Following three washes in 1× PBS cell nuclei were labeled using Hoechst 33258 (Invitrogen, H3569). Cells were imaged using a confocal microscope (Zeiss, LSM 880 Airyscan).

### Depletion of *Cx3cr1*^+^ cells

Tamoxifen (Sigma, T5648) was resuspended in corn oil (Sigma, C8267) and heated to 75°C in a water bath and kept at 75°C for 60 min. The Cre-recombinase in CX3CR1^CreER/+^Rosa26^DTA/+^ mice was activated by injecting 5 mg (200 µl) tamoxifen subcutaneously once daily for a total of 3 d. The depletion was evaluated and confirmed at various time points. Therefore, animals treated with tamoxifen were killed and the spinal cords harvested as described above. The spinal cords were stained and surface marker expression analyzed using flow cytometry (Beckman Coulter, Gallios). Data were analyzed using Kaluza software (Beckman Coulter). A piece of each spinal cord was preserved for histologic evaluation and was evaluated as described above.

### Evaluation of hind limb locomotor function

The recovery of hindlimb locomotor function following SCI was evaluated using the Basso mouse scale (BMS; Basso et al., 2006) before, immediately following, and then weekly post-SCI until 90 d following SCI. Two researchers discussed and assessed the score. The mice were evaluated in a custom-made open-field environment constructed using Plexiglas.

### Bulk RNAseq of microglia/macrophages

#### FACS

Microglia/macrophages were defined as CX3CR1^+^CD19^–^Ly6G^–^CD3^–^NK1.1^–^SIGLECF^–^CD11b^+^CD45^high/low^ cells and sorted into FACS buffer using a BD Influx cell sorter.

#### Isolation of total RNA

Sorted microglia/macrophages in FACS buffer were collected by centrifugation at 300 × *g* for 5 min and resuspended in 1-ml TRIzol reagent (Thermo Fisher Scientific, 15596026) and stored at −70°C until downstream analysis. RNA was isolated using the manufacturer's (TRIzol) protocol. Isopropanol precipitation was conducted using 75-µg glycogen (GlycoBlue Coprecipitant, Thermo Fisher Scientific AM9516) at −20°C O/N. RNA clean-up was performed using RNeasy micro kit (QIAGEN, 74004) according to the manufacturer's protocol. Contaminating genomic DNA was removed during the isolation by on-column digestion with DNase (DNase I QIAGEN, 79254). RNA was stored at −70°C.

#### Library preparation, sequencing, and differential gene expression analysis

Total RNA from microglia/macrophages was sequenced. Sequencing libraries were prepared using SMARTer Stranded Total RNA-Seq kit-Pico Input Mammalian (Clontech) because of the low amount of RNA. Libraries were sequenced 2 × 125 bp on three lanes using HiSeq2500 (Illumina Inc.) on high output mode. For each replicate a minimum of 15 × 10^6^ read-pairs were used. TrimGalore (Babraham Bioinformatics) was used for removal of low quality regions and adapter sequences. Read-pairs were aligned to the mouse genome (GRCm38, build mm10) using STAR (Table 5). featureCounts (Liao et al., 2014) and Ensembl annotation release 81 was used for summary of read counts over genes. Differential gene expression analysis was conducted in R (v3.6.0; Team} RC, 2016) using limma (Ritchie et al., 2015) and edgeR (Robinson et al., 2010) with annotations from Mus.musculus (Team BC, 2015).

### Proliferation analysis

Spinal cords subjected to SCI were dissociated and stained with antibody against CD45 as described above. Following wash, the cells were incubated in 1:2000 Hoechst 33342 for 15 min, washed twice in 1× DPBS and resuspended in 0.5 ml 1× DPBS. Live/dead marker was added to the suspension in the same manner as described above (Table 2). Surface expression data were collected and analyzed offline. The CD45^+^ cells were divided into CD45^low^ and CD45^high^. For each subset (low/high) the expression of Hoechst 33342 (Thermo Fisher Scientific, 62249) was plotted in a histogram. The cells with the highest Hoechst 33342 expression were defined as cells in S-phase of the cell cycle and therefore proliferative cells.

### Statistical analysis

Data for hindlimb function, proliferation rate and number of microglia following depletion was presented for each biological replicate in combination with a mean surrounded by a 95% confidence interval (CI). Hind limb function was further investigated using a mixed ANOVA followed by Wilcoxon *post hoc* test between groups within each time point. Correlation between current data and peer data were conducted using Pearson's product moment correlation coefficient.

## Results

### Microglia undergo rapid and profound changes on SCI, ultimately arresting in a chronic DAM-like state

**Figure 1.**
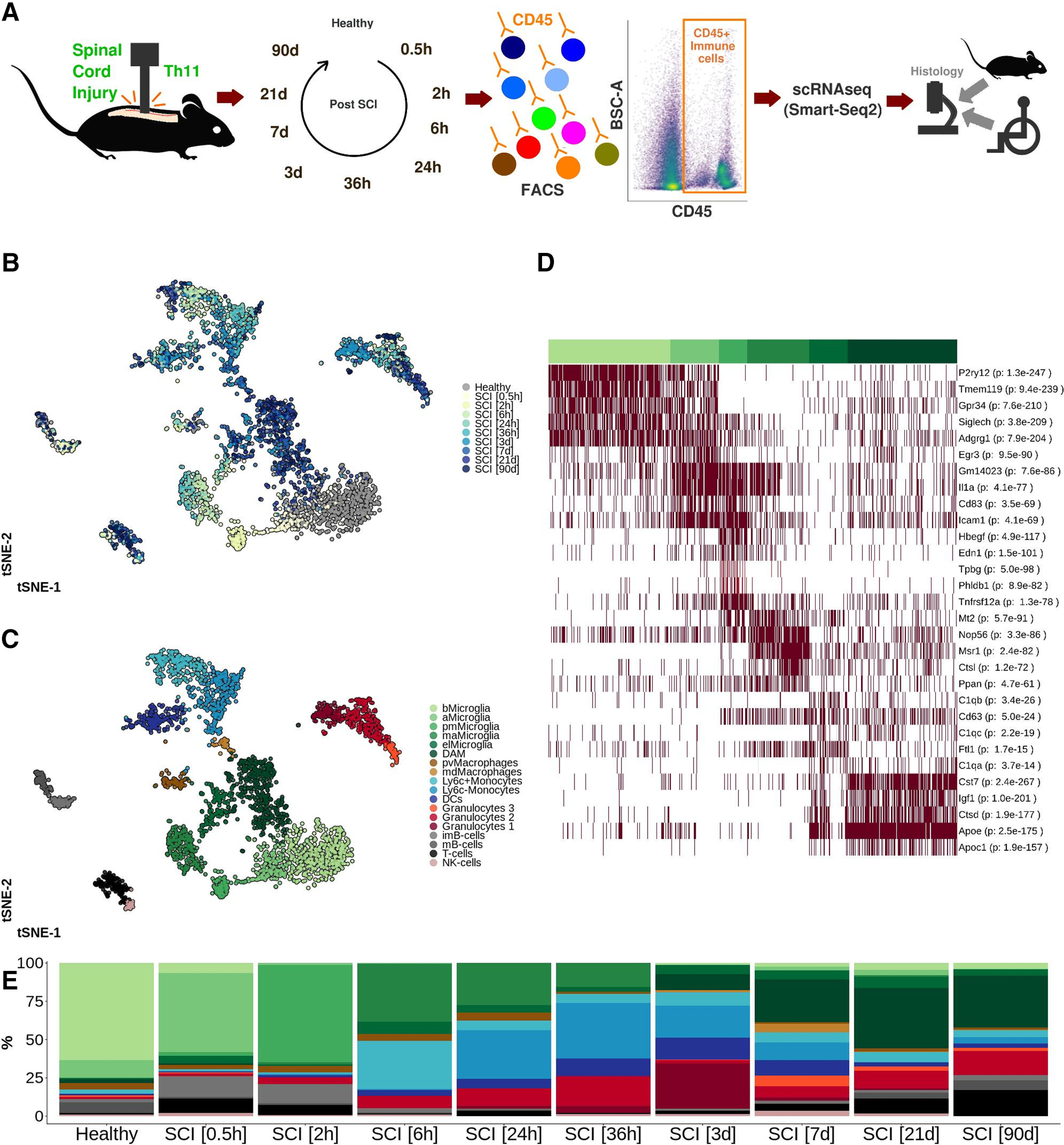
***A***, Severe contusion SCI was induced in the thoracic spinal cord of adult female wild-type mice. CD45^+^ immune cells from the SCI epicenter were isolated using FACS and sequenced individually following library preparation using the Smart-Seq2 protocol. Thin sections from mouse and human spinal cord were subjected to histologic analysis. ***B***, tSNE plot of 3069 individually sequenced CD45^+^ immune cells from SCI. Healthy spinal cord tissue was used as reference. Color of the cell corresponds to the time point following SCI at which the spinal cord was harvested and evaluated. ***C***, tSNE plot of 3069 CD45^+^ immune cells from mouse SCI. Eighteen clusters were detected and annotated using hallmark genes. bMicroglia: baseline microglia; aMicroglia: activated microglia; pmMicroglia: proliferation-mediating microglia; maMicroglia: monocyte-activating microglia; DAM: disease-associated microglia in SCI; elMicroglia: embryonic-like microglia, pvMacrophages: perivascular macrophages; mdMacrophages: monocyte-derived macrophages; DCs: dendritic cells; imB-cells: immature B-cells; mB-cells: mature B-cells. ***D***, Heatmap representation of row-wise normalized log2(cpm) expression for the top five marker genes (ranked on FDR) for microglial cell clusters detected in ***A***. Each row represents one gene and each column represents one cell. Clusters are annotated using color-coding as in ***A***. *p* value adjusted for multiple comparisons indicating significance of marker gene is reported in parenthesis. ***E***, Bar plot reporting the percentage portion which each cell type makes up out of all cells at each time point of evaluation. This figure is extended in Extended Data Figures 1-1, 1-2, 1-3, 1-4.

We characterized the immune cell population over time following severe traumatic contusion SCI in mice. CD45^+^ cells were isolated from the injury epicenter (site of impact ±3 mm along the cranial-caudal axis), and libraries for each individual cell were prepared using the Smart-Seq2 protocol (Picelli et al., 2014). To ensure that the time-points were comparable and to minimize the impact of random variation, a pooling strategy was implemented in which a suspension of cells collected from spinal cords of three to five mice were used for each time point. The immune cell response was investigated at nine time points following SCI, spanning both the acute and chronic phase, and using healthy spinal cord as reference (Fig. 1*A*). A total of 3069 individually sequenced immune cells were included following quality control (Extended Data Fig. 1-1*A–G*; Table 7). The cells organized into distinct clusters, with various degrees of temporal influence (Fig. 1*B*), whereas the transcriptional profile of B and T cells remained largely constant over time, microglia underwent a dramatic transformation following SCI, and never reestablished their original transcriptional profile at any time point following SCI (Extended Data Fig. 1-2*A*,*B*).

**Table 7.** Quality control of sequenced cells

				Cells (*n*)			
Sequenced				4734			
(–) low read count				888			
(–) low Actb expression				159			
= included in analysis				3687			
Plate ID	Sort gate	Condition	Cells sequenced (*n*)	Cells with low read count (*n*)	Cells with low *Actb* expression (*n*)	Cells which passed QC (*n*)	Cells which passed QC (%)
VZC00104	CD45^+^	Healthy	384	22	7	355	92.45
VZC00214		Healthy	160	22	2	136	85.00
VZC00215		Healthy	160	30	1	129	80.62
VZC00703		SCI (0.5 h)	192	38	3	151	78.65
VZC00801		SCI (2 h)	191	31	6	154	80.63
VZC00601		SCI (6 h)	384	70	4	310	80.73
VZC00604		SCI (24 h)	384	93	10	281	73.18
VZC00803		SCI (36 h)	192	38	0	154	80.21
VZC00501		SCI (3 d)	384	47	6	331	86.20
VZC00101		SCI (7 d)	384	55	15	314	81.77
VZC00205		SCI (7 d)	384	108	13	263	68.49
VZC00401		SCI (21 d)	384	127	14	243	63.28
VZC00901		SCI (90 d)	383	42	77	264	68.93
VZC01001		SCI MD (7 d)	384	71	2	311	80.99
VZC01101		SCI MD (21 d)	384	74	17	293	76.30

MD: microglia (Cx3cr1^+^ cell) depletion; QC: quality control. h: hour; d: day; n: number.

10.1523/JNEUROSCI.0860-21.2021.f1-1 Extended Data Figure 1-1 ***A***, Histogram (100 bins) of log2(cpm) of *Actb* expression in each cell. Red line indicates the 1st percentile. All cells with *Actb* expression > 1st percentile threshold were included in the analysis. ***B***, log2-log2 plot of number of features and total number of counts per cell. Red line indicates a minimum total count of 2^16^ per cell. All cells with a total count exceeding 2^16^ were included in the analysis. ***C***, Scaled dispersion for each gene plotted against the mean log2(cpm) expression for each gene. A total 1168 genes indicated with red color were selected as highly variable genes and used in the downstream analysis pipeline. ***D***, Standard deviation for the first 50 PCs. Red line indicates the 17 first components, which were deemed to be the most variable components and therefore included in the *FindNeighbors()* function when clustering cells. ***E***, tSNE plot of 3069 CD45^+^ immune cells which passed the quality control. Number of features per cell is indicated using a continuous color. ***F***, Equivalent to ***E*** and indicates the number of genes with a positive value for log2(count) per cell using a continuous color. ***G***, Histograms of the total number of counts per cell for each 384-well plate included in the analysis. Red line indicates the 2^16^ threshold determined in ***B***. Download Figure 1-1, TIF file.

**Figure 2.**
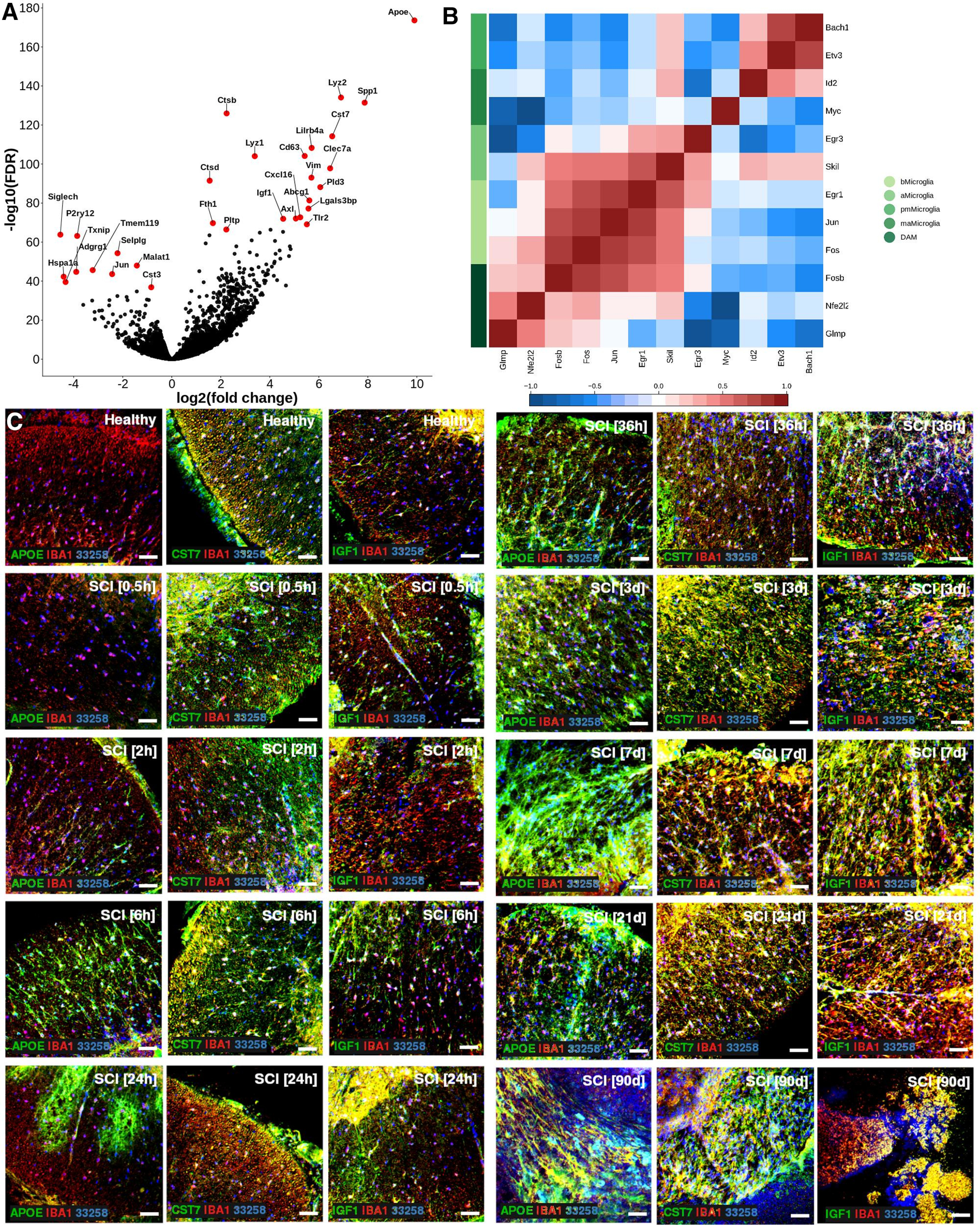
***A***, Volcano plot depicting differentially expressed genes between DAM in SCI and baseline microglia. Top 15 significantly upregulated genes and top 10 significantly downregulated genes are indicated in red and with gene symbol. ***B***, Correlogram of top (maximum 3) unique transcription factors for each microglial cell type. Cell type is indicated using color. Lower color key indicates the direction and size of the correlation. bMicroglia: baseline microglia; aMicroglia: activated microglia; pmMicroglia: proliferation-mediating microglia; maMicroglia: monocyte-activating microglia; DAM: disease-associated microglia in SCI; elMicroglia: embryonic-like microglia. ***C***, Protein expression for APOE, CST7, and IGF1 on mouse spinal cord sections. Sections are taken just rostral to SCI epicenter. Scale bars: 50 µm.

10.1523/JNEUROSCI.0860-21.2021.f1-2 Extended Data Figure 1-2 ***A***, tSNE plots of 3069 CD45^+^ immune cells in SCI. Each plot corresponds to a time point post-SCI. Healthy tissue is used as reference. ***B***, Proportion of cells within each cell type associated with a specific time point of evaluation. Healthy tissue is used as reference. Download Figure 1-2, TIF file.

**Figure 3.**
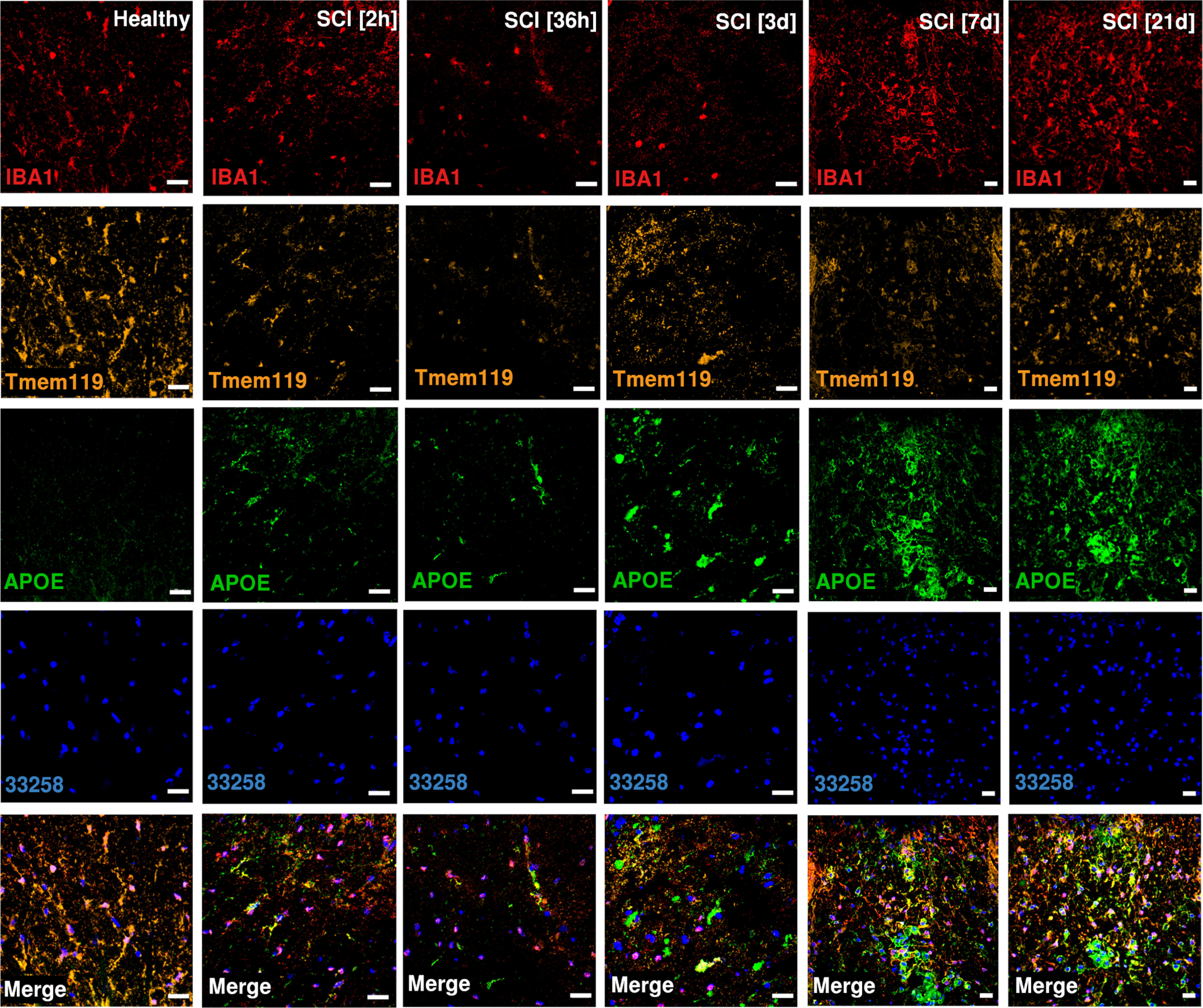
Protein expression for APOE, IBA1 and TMEM119 on mouse spinal cord sections. Sections are taken just rostral to SCI epicenter. Scale bars: 20 µm.

10.1523/JNEUROSCI.0860-21.2021.f1-3 Extended Data Figure 1-3 ***A***, tSNE plots of 3069 CD45^+^ immune cells following index sorting using surface markers CD11b, CD45, CD14, and CD64. Log2(fluorescence) is represented with a continuous color. ***B***, FACS plots of CD45 surface expression versus BSC-A for each 384-well plate separately (time point post-SCI indicated in plot). Leftmost FACS plots are density plots reporting all analyzed cells. Rightmost plots are equivalent to leftmost plots with sorted and sequenced cells annotated based on cell type using color. bMicroglia: baseline microglia; aMicroglia: activated microglia; pmMicroglia: proliferation-mediating microglia; maMicroglia: monocyte-activating microglia; DAM: disease-associated microglia in SCI; elMicroglia: embryonic-like microglia; pvMacrophages: perivascular macrophages; mdMacrophages: monocyte-derived macrophages; DCs: dendritic cells; imB-cells: immature B-cells; mB-cells: mature B-cells. Download Figure 1-3, TIF file.

**Figure 4.**
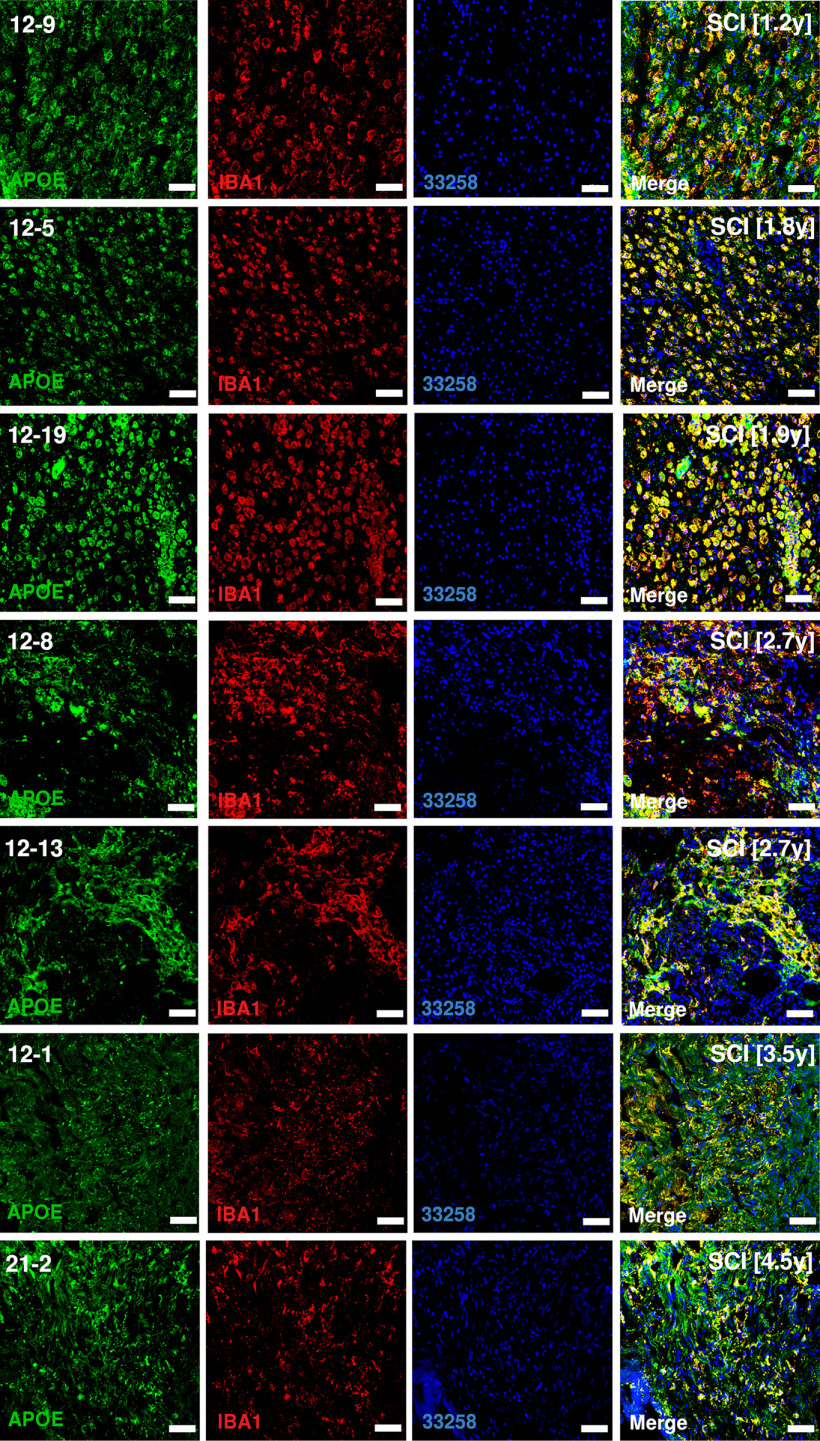
Protein expression of APOE on thin sections of human SCI samples. Sections are rostral to SCI epicenter. Number in brackets reports the number of years from SCI to tissue harvest. Scale bars: 50 µm.

10.1523/JNEUROSCI.0860-21.2021.f1-4 Extended Data Figure 1-4 ***A***, CX3CR1^+^CD19^–^Ly6G^–^CD3^–^NK1.1^–^SIGLECF^–^CD11b^+^CD45^high/low^ cells were sorted from SCI from which RNA was sequenced in bulk at 7 d (*n* = 3) and 21 d (*n* = 3) post-SCI. Healthy tissue was used as reference (*n* = 4). Sorted cells were plated and cultured for 72 h and stained for IBA1. ***B***, Agglomerative hierarchical clustering with heat map representation of biological replicates for 3323 significantly differentially expressed genes (FDR < 0.01; logFC<-1 OR logFC > 1) for all three contrasts. Condition indicated with color and reported in ***C***. ***C***, log2(cpm) expression for the top 15 upregulated and downregulated genes in DAM in SCI and baseline microglia (bMicroglia). Download Figure 1-4, TIF file.

**Figure 5.**
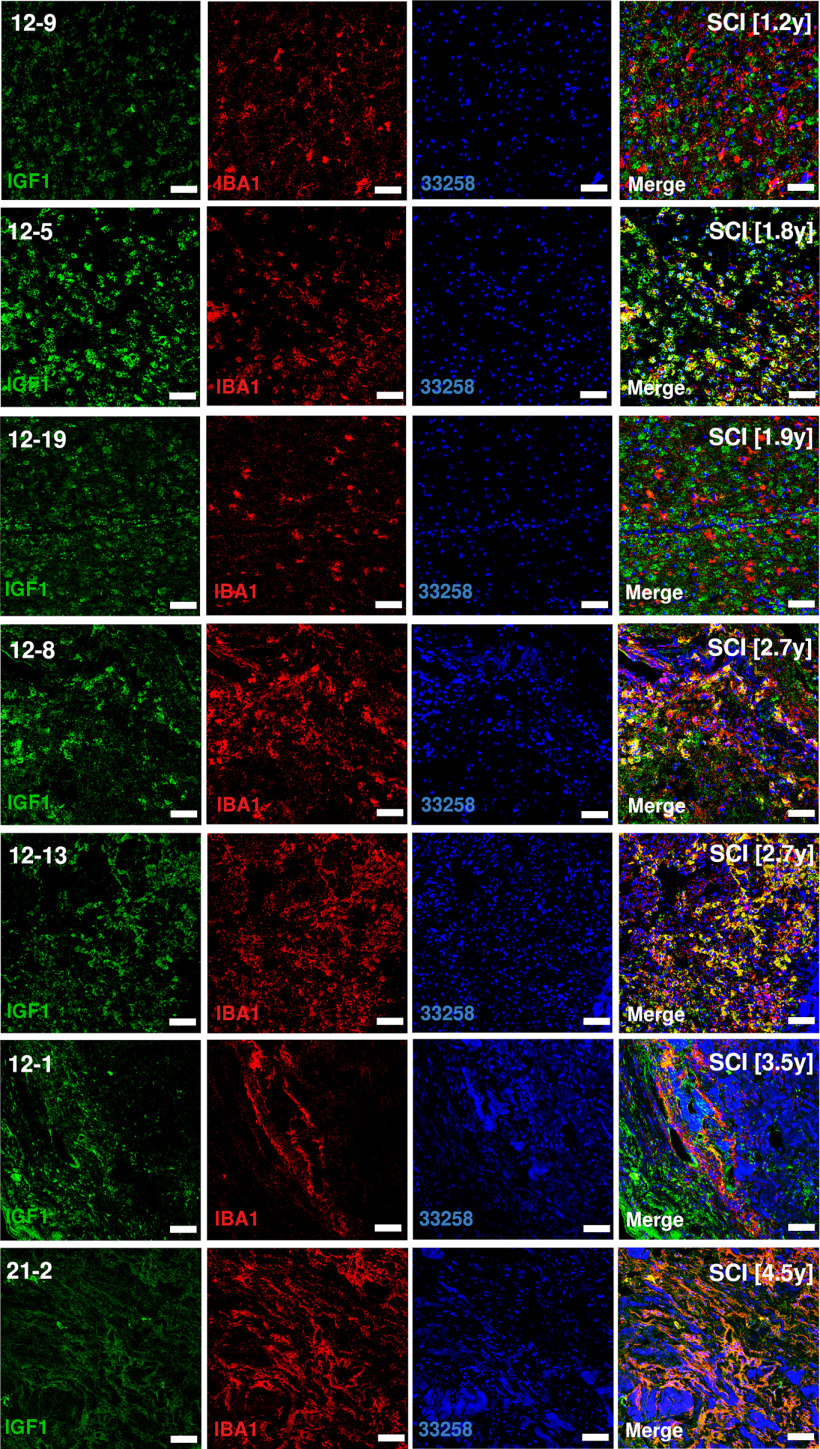
Protein expression of IGF1 on thin sections of human SCI samples. Sections are rostral to SCI epicenter. Number in brackets reports the number of years from SCI to tissue harvest. Scale bars: 50 µm.

**Figure 6.**
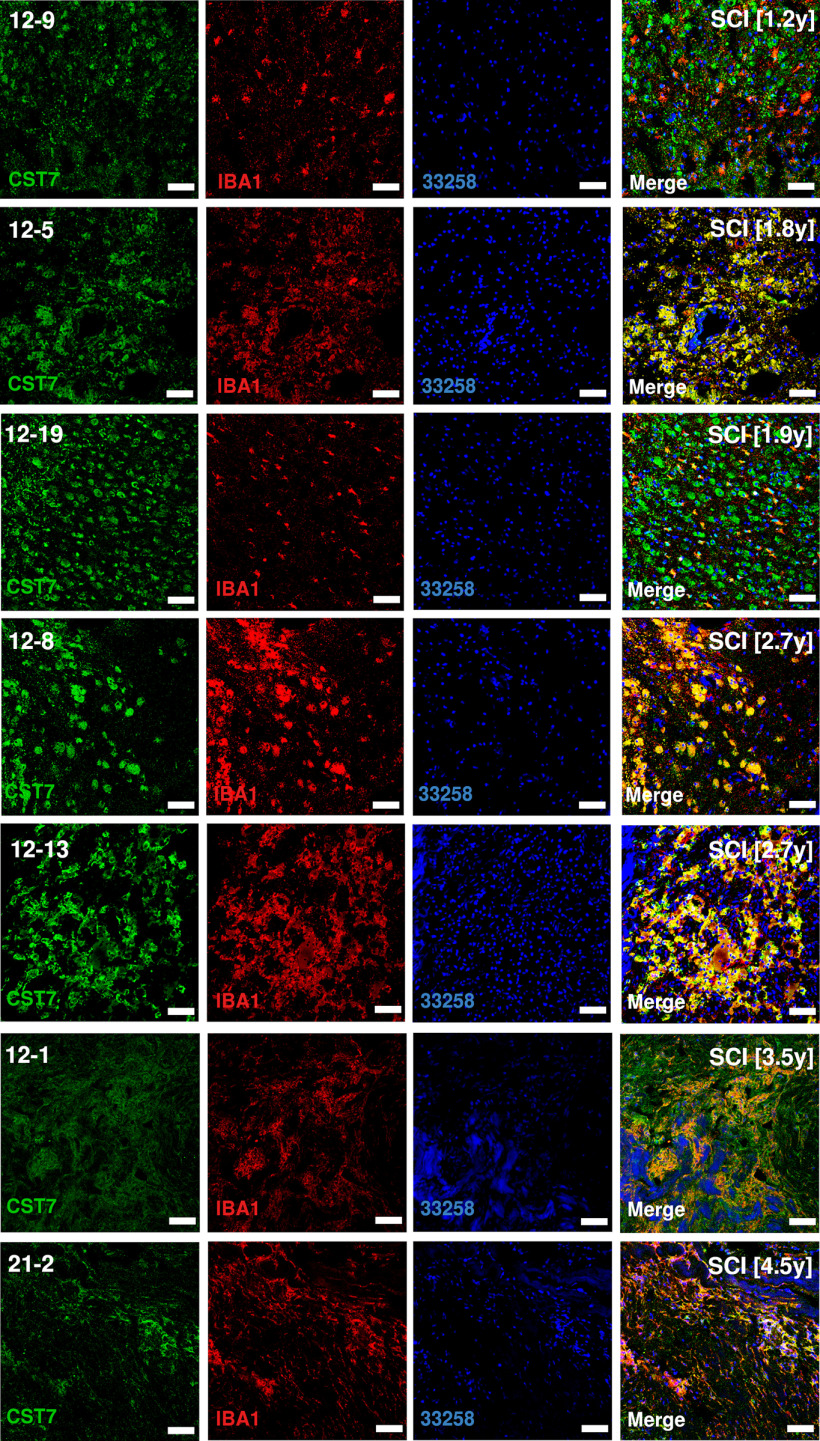
Protein expression of CST7 on thin sections of human SCI samples. Sections are rostral to SCI epicenter. Number in brackets reports the number of years from SCI to tissue harvest. Scale bars: 50 µm.

Five subtypes of microglia were identified (Fig. 1*C*; Extended Data Fig. 1-2*B*). Baseline microglia (bMicroglia) defined by expression of *P2ry12, Tmem119* and *Siglech* underwent a rapid transformation into an activated form of microglia (aMicroglia), defined by expression of inflammation-associated markers such as *Il1a* and *Cd83*, already at 0.5 h post-SCI (Fig. 1*C–E*). aMicroglia had fully transformed into a distinct population of proliferation-mediating microglia (pmMicroglia) defined by expression of the growth factor *Hbegf* as well as *Edn1* at 2 h post-SCI. The transformation halted between 6 and 36 h in a state defined by expression of *Mt2* and *Msr1* resulting in monocyte-activating microglia (maMicroglia). DAM-like cells appeared at 3 d post-SCI, and increased in number from 3 to 21 d post-SCI when it reached a steady state which persisted during the chronic phase of SCI (90 d post-SCI; Fig. 1*C–E*; Extended Data Fig. 1-2*A*). These cells bear all the hallmarks of DAM described in mouse models of neurodegeneration, demyelination and development (Wang et al., 2015; Keren-Shaul et al., 2017; Krasemann et al., 2017; Mathys et al., 2017; Friedman et al., 2018; Mrdjen et al., 2018; Olah et al., 2018; Sousa et al., 2018; Tay et al., 2018; Hammond et al., 2019; Jordão et al., 2019; Li et al., 2019; Masuda et al., 2019; O'Koren et al., 2019; Figs. 1*D*, 2*A*). A distinct set of transcription factors could be determined for each microglial subtype, with *Egr3* and *Skil* associated with aMicroglia, pmMicroglia mainly associated with *Bach1* and *Etv3*, while maMicroglia were associated with transcription factors *Id2* and *Myc*. *Glmp* and *Nfe2l2* were transcription factors unique for DAM in SCI, while *Fosb* was expressed in both DAM and bMicroglia. Similarly, *Skil* was expressed in both aMicroglia and bMicroglia (Fig. 2*B*). Protein expression of CST7, IGF1, and APOE was confirmed using immunohistochemistry on thin sections of injured spinal cord from both mice and humans (Figs. 2*C*, 3–6). Microglia and DAM could not be distinguished using surface expression of CD45 alone (Extended Data Fig. 1-3), but differential expression of the genes defining the two cell types could be detected in bulk RNAseq (Extended Data Fig. 1-4).

**Figure 7.**
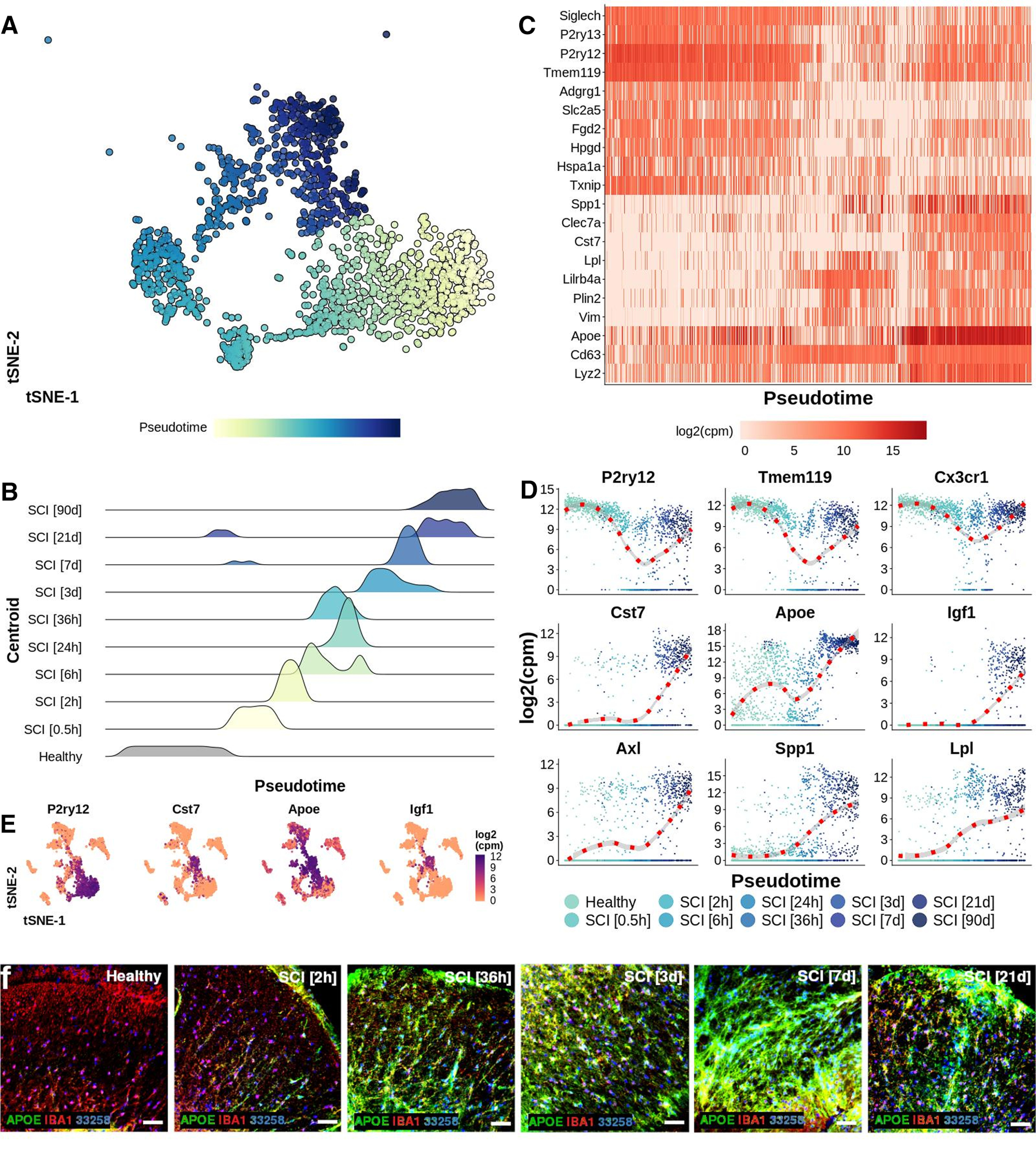
***A***, tSNE plot of 1474 microglial cells arranged along pseudotime. Cells were ordered along pseudotime by estimating a principal curve using centroids for each condition as anchors. Centroids were estimated by calculating the mean tSNE-1/2 value for all cells within each condition. Each cell was then assigned to a centroid based on a minimum Euclidean distance approach. ***B***, Density of 1474 microglial cells in each centroid along pseudotime. ***C***, Heat map reporting log2(cpm) expression for selected genes for 1474 microglial cells ordered along pseudotime. A linear model was estimated for all ∼20,000 genes. The 10 genes with the most positive slopes and the 10 genes with the most negative slopes were included in the heat map. The genes were ordered vertically using hierarchical clustering. Rows represent genes while columns represent cells. ***D***, log2(cpm) expression for microglial cells for selected genes arranged along pseudotime. A locally estimated scatterplot smoothing (loess) was estimated for each gene and reported using a red dotted line in combination with a 95% CI. Each dot represents one cell. ***E***, tSNE plots of 3069 CD45^+^ immune cells reporting the log2(cpm) expression with a continuous color for selected genes. ***F***, Protein expression of APOE on mouse spinal cord sections. Scale bars: 50 µm.

Pseudotime analysis of the microglial populations confirmed our initial classification (Fig. 7*A*), with bMicroglia undergoing a rapid transformation immediately following SCI (0–2 h), adopting an intermediate state between 6 and 36 h and gradually falling into the steady-state DAM profile which persisted in the chronic phase of the injury (90 d post-SCI; Fig. 7*B*). Genes defining bMicroglia were downregulated over pseudotime, while the opposite was true for genes defining DAM in SCI (Figs. 2*A*, 7*C–E*). Protein expression of CST7, IGF1, and APOE concorded with the gene expression, both in mouse and human tissue sections (Figs. 2*C*, 3–6, 7*F*).

Taken together, our results show a rapid transformation of microglial cells following injury, in combination with a loss of baseline markers and gradual adoption of a permanent disease-associated cell state. DAM in SCI appear to be low-proliferative microglial cells defined by genes related to lipoprotein and cholesterol metabolism, lysosomal proteolysis as well as cell and organelle adhesion. In combination with the activation of distinct transcription factor cascades, this suggests transcriptional reprogramming of bMicroglia into DAM, via a previously unrecognized intermediate state during the first week post-SCI. Interestingly, this intermediate state was more distinct from bMicroglia than chronic DAM.

### DAM in trauma, demyelination, degeneration, and development of the CNS are transcriptionally similar

**Figure 8.**
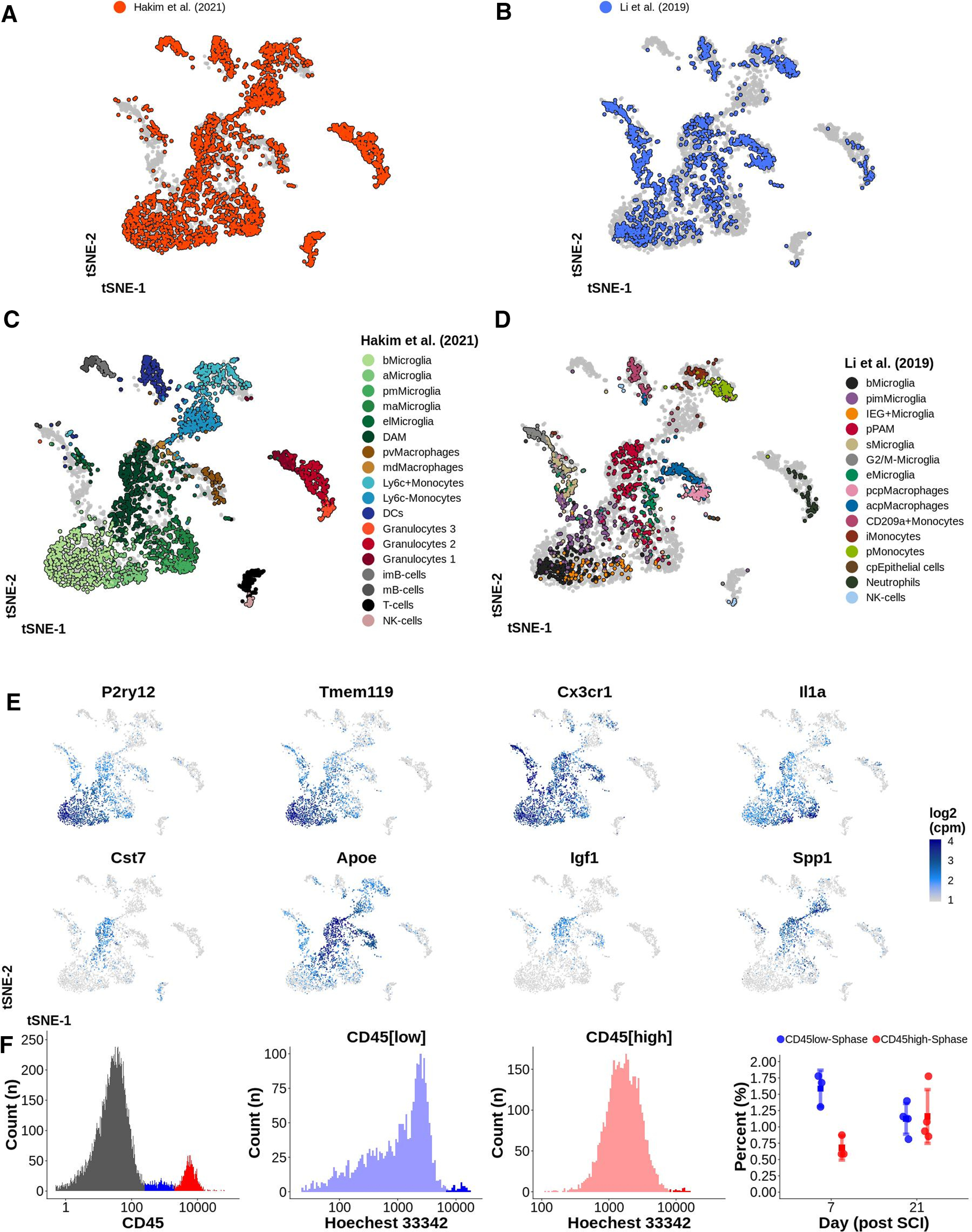
***A***, ***B***, tSNE plot of 4659 CD45^+^ immune cells from the CNS. Data from this study is integrated with data from Li et al. (2019; Table 6). ***C***, ***D***, Each dataset represented by one tSNE. Cells are annotated using color representing their cell type. bMicroglia: baseline microglia; aMicroglia: activated microglia; pmMicroglia: proliferation-mediating microglia; maMicroglia: monocyte-activating microglia; DAM: disease-associated microglia in SCI; elMicroglia: embryonic-like microglia; pvMacrophages: perivascular macrophages; mdMacrophages: monocyte-derived macrophages; DCs: dendritic cells; imB-cells: immature B-cells; mB-cells: mature B-cells; pimMicroglia: postnatal immature microglia; IEG+Microglia: immediate early genes microglia; sMicroglia: s-phase microglia; pPAM: postnatal proliferative-region-associated microglia; eMicroglia: embryonic microglia; pcpMacrophages: postnatal choroid plexus macrophages; acpMacrophages: adult choroid plexus macrophages; iMonocytes: inflammatory monocytes; pMonocytes: patrolling monocytes; cpEpithelial cells: choroid plexus epithelial cells. ***E***, tSNE plots of 4659 CD45^+^ immune cells for selected genes indicating the log2(cpm) expression with a continuous color for each cell separately. ***F***, Proliferation rate of CD45^low^ and CD45^high^ cells at 7 and 21 d post-SCI. Hoechst 33342 expression presented on a log-scale was used for defining cells in S-phase. Right most plot reports the percentage of cells in S-phase. Each dot represents one biological replicate (four mice per time point). Mean is surrounded by a 95% CI. This figure is extended in Extended Data Figure 8-1.

Based on marker gene expression, we found that DAM in SCI bore a striking similarity to DAM found during neurodegeneration, demyelination and development. In order to more coherently compare our cells to those found in other disease processes, we used molecular anchor-based integration of our dataset with published datasets. Li et al. (2019) sequenced 2123 microglia and myeloid cells from mouse brain during three different stages of development (Table 6), resulting in a dataset of 4659 immune cells prepared using similar methodology to our work (Fig. 8*A*,*B*). The authors detected postnatal proliferative-region-associated microglia (pPAM), a subtype of microglia with striking similarities to DAM, embedded in the white matter of developing mouse brains. pPAM were metabolically active with an amoeboid morphology and phagocytized newly formed oligodendrocytes. As expected, DAM in SCI co-localized with pPAM in the aggregated tSNE (Fig. 8*C*,*D*). aMicroglia in SCI co-localized with immediate early genes (IEG)+Microglia described by Li et al., while cells expressing high levels of *C1qa-c* overlapped with embryonic microglia (eMicroglia) and were therefore termed embryonic-like microglia (elMicroglia; Fig. 8*E*). The proliferative cluster (G2/M-microglia) found in embryonal tissue, was however missing from our data, which is consistent with our observed low rate of proliferation (∼1.0–1.5% of all CD45^low^ cells were in S-phase). No significant difference in proliferation was detected at 7 and 21 d post-SCI (Fig. 8*F*). Integration of datasets thus confirmed the similarities between microglial subtypes in traumatic injury to a subtype of microglia found in developing brain.

10.1523/JNEUROSCI.0860-21.2021.f8-1 Extended Data Figure 8-1 ***A***, tSNE plot of 10014 immune and microglial cells following integration of the data in this study with three published datasets. ***B***, Cells from one study annotated in each tSNE using the original metadata. bMicroglia: baseline microglia; aMicroglia: activated microglia; pmMicroglia: proliferation-mediating microglia; maMicroglia: monocyte-activating microglia; DAM: disease-associated microglia in SCI; elMicroglia: embryonic-like microglia; pvMacrophages: perivascular macrophages; mdMacrophages: monocyte-derived macrophages; DCs: dendritic cells; imB-cells: immature B-cells; mB-cells: mature B-cells; pimMicroglia: postnatal immature microglia; IEG+Microglia: immediate early genes microglia; sMicroglia: s-phase microglia; pPAM: postnatal proliferative-region-associated microglia; eMicroglia: embryonic microglia; pcpMacrophages: postnatal choroid plexus macrophages; acpMacrophages: adult choroid plexus macrophages; iMonocytes: inflammatory monocytes; pMonocytes: patrolling monocytes; cpEpithelial cells: choroid plexus epithelial cells. ***C***, tSNE plots of 10014 immune and microglial cells reporting log2(cpm) expression of selected genes with a continuous color for each cell separately. ***D***, logFC for contrast DAM versus bMicroglia correlated between SCI, pPAM, and AD. Download Figure 8-1, TIF file.

We similarly analyzed two published datasets collected from models of neurodegenerative disease in the CNS (10,014 cells in total; Table 6). Masuda et al. (2019) used scRNAseq to investigate microglia in different regions of the brain when using a model of toxic demyelination (cuprizone) and neurodegeneration (unilateral n.VII axotomy). We integrated 3721 microglia harvested from healthy tissue as well as from brain and spinal cord tissue subjected to n.VII axotomy or demyelination into our dataset (Extended Data Fig. 8-1*A*). In the aggregated tSNE microglia during demyelination co-localized somewhat with DAM in SCI, while microglia following n.VII axotomy (3/14 d after injury) did not co-localize (Extended Data Fig. 8-1*B*,*C*). Microglia harvested from healthy 3- and 16-week-old mice were located in proximity to, but did not overlap with bMicroglia. However, embryonal microglia (healthy) formed a distinct cluster in proximity to DAM and pPAM. Thus, microglia during injury seem to have a transcriptional profile similar to embryonal and postnatal microglia. The differences between bMicroglia and microglia at 3 and 16 weeks might be explained by Masuda et al. (2019): (1) using CD-1 mice as compared with C57BL/6J mice in this study; (2) using both brain and spinal cord tissue as compared with spinal cord only in this study; (3) gating, sorting and sequencing selected microglia (CD45^int^CD11b^+^Ly6C^–^Ly6G^–^CD206^–^) as compared with the current study which investigated all CD45^+^ cells/microglia and d) evaluating microglia during de/remyelinzation in the corpus callosum only. Mathys et al. (2017) isolated and sequenced 1634 microglial cells from the hippocampus of mice with neurodegeneration resulting in symptoms of AD (CK-p25 inducible mice). The microglial cells from these CK-p25 mice broadly matched our DAM, but no clear subtype co-localization was detected (Extended Data Fig. 8-1*A–C*). Correlation analysis using Pearson's product moment correlation coefficient between differential gene expression for DAM in SCI and pPAM and microglia during AD (Mathys et al., 2017), respectively, confirmed the downregulation of baseline genes and upregulation of disease-associated genes (Extended Data Fig. 8-1*D*). Taken together, our analysis strongly supports that DAM in trauma, demyelination and development are similar both with respect to expression of effector genes, whole transcriptional profile, and morphology (where investigated).

### DAM in SCI are permanently reprogrammed baseline microglia which enhance functional recovery

Having established the identity of DAMs in the chronic phase SCI response, two important questions remain. First, do DAM cells originate from resident baseline microglia or peripheral infiltrating cells? Our pseudotime analysis strongly suggest that bMicroglia gradually change states to transition into DAM cells, but does not formally exclude a peripheral origin from tissue or bone marrow reservoirs. Second, do DAM cells impact functional recovery following SCI? Previous studies have used disease models in which the evaluation of the function is difficult, in contrast to gross motor evaluation in a SCI model.

**Figure 9.**
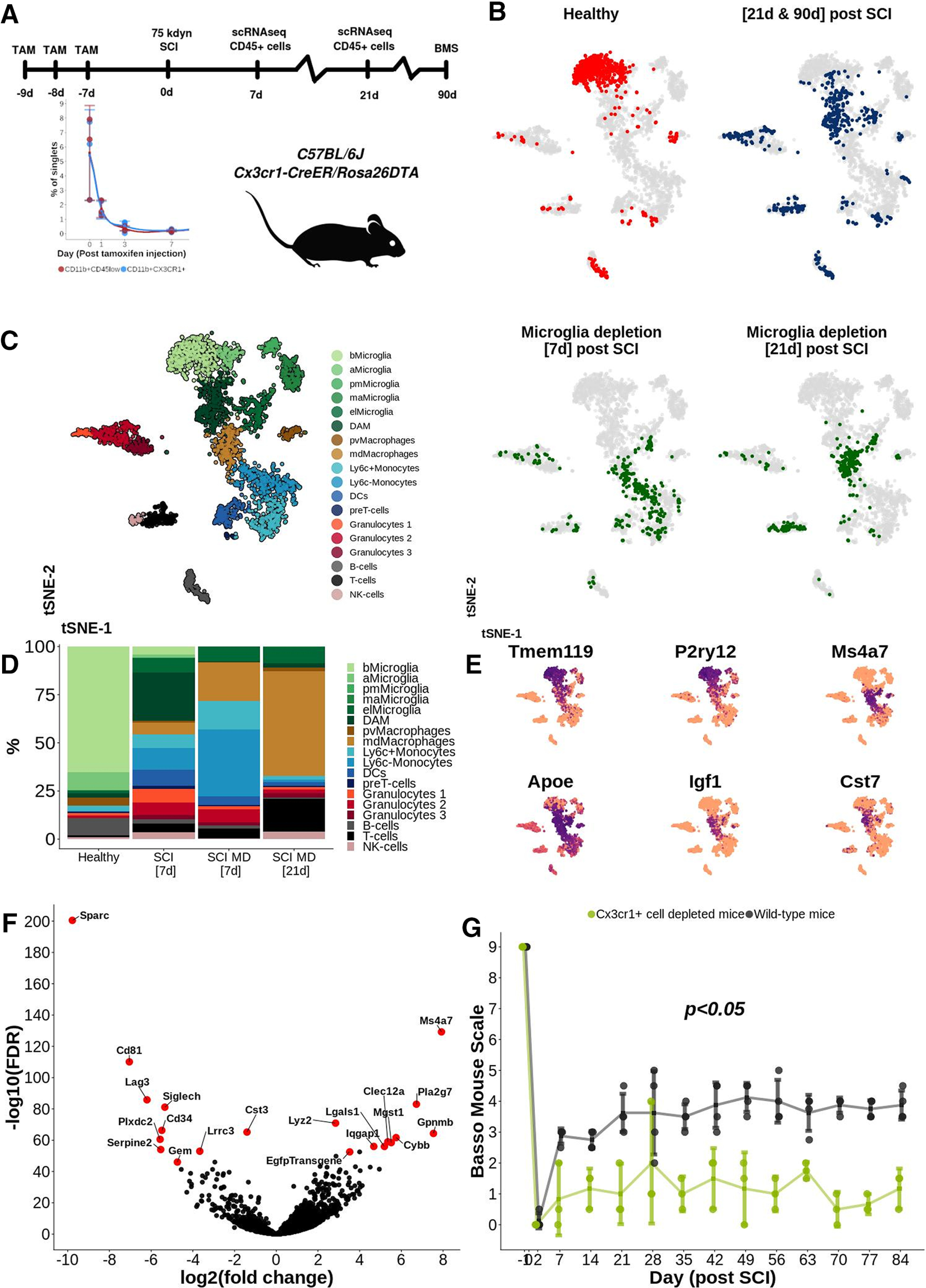
***A***, Experimental design. TAM: tamoxifen. ***B***, tSNE plot of 3687 CD45^+^ immune cells. Color indicates treatment and time point of evaluation. Cells at 21 and 90 d post-SCI are labeled using the same color in the same tSNE. Microglia depletion refers to CD45^+^ immune cells isolated from mice subjected to microglial depletion before SCI. ***C***, tSNE plot of 3687 CD45^+^ immune cells clustered and annotated according to cell type. bMicroglia: baseline microglia; aMicroglia: activated microglia; pmMicroglia: proliferation-mediating microglia; maMicroglia: monocyte-activating microglia; DAM: disease-associated microglia in SCI; elMicroglia: embryonic-like microglia, pvMacrophages: perivascular macrophages; mdMacrophages: monocyte-derived macrophages; DCs: dendritic cells; imB-cells: immature B-cells; mB-cells: mature B-cells. ***D***, Bar plot reporting the percentage portion which each cell type makes up out of all cells at each time point of evaluation. ***E***, tSNE plot of 3687 CD45^+^ immune cells reporting log2(cpm) expression for a set of selected genes using a continuous color for each cell separately. ***F***, Volcano plot reporting differentially expressed genes between mdMacrophages and bMicroglia. ***G***, BMS score over time. Each dot represents one animal. Mean value for each time point and study group is surrounded by a 95% CI; *p < 0.05; ns, not significant.

**Figure 10.**
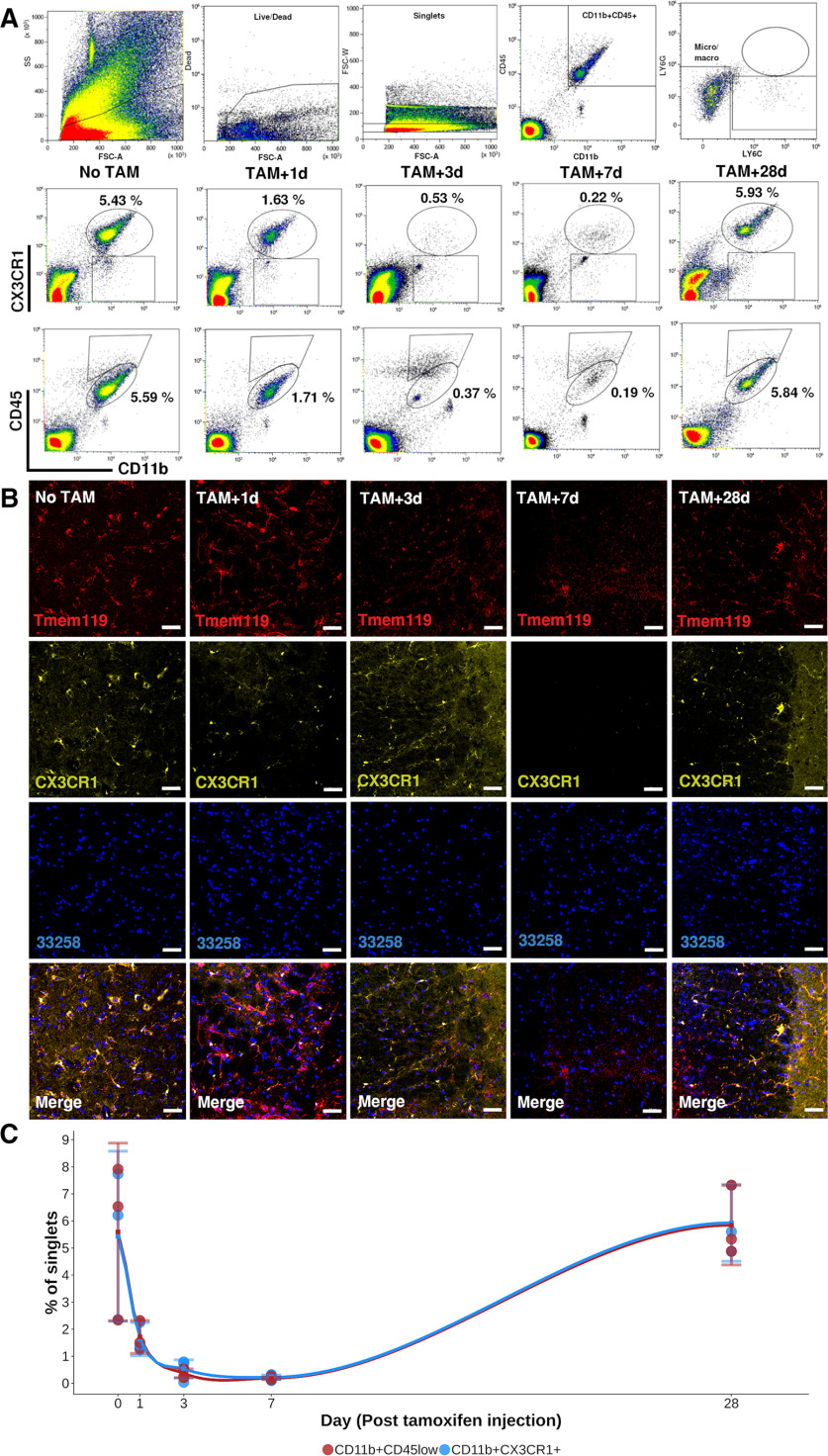
***A***, Upper row depicts the gating strategy of spinal cord cells from *CX3CR1*^*CreER*/+^*Rosa26*^*DTA*/+^ mice injected with tamoxifen (TAM). Second and third row presents representative FACS plots for expression of CD11b, CD45, and CX3CR1 at 1, 3, 7, and 28 d after last TAM injection. Microglia is defined as CD11b^+^CD45^low^ or CD11b^+^CX3CR1^+^ cells. Average percentage of singlets in the specified gates are reported in the plots. ***B***, Protein expression of TMEM119 and CX3CR1 on representative thin histologic sections of spinal cord at each time point. ***C***, Level of microglia in the spinal cord over time following injection of TAM presented as percentage of singlets in the CD11b^+^CD45^low^ and CD11b^+^CX3CR1^+^ gates, respectively. Each dot represents one animal. Mean value at each time point of evaluation is surrounded by a 95% CI.

To address these questions, we used a transgenic mouse model (CX3CR1^CreER/+^Rosa26^DTA/+^), which allows tamoxifen-dependent conditional depletion of Cx3cr1^+^ cells. Cx3cr1 is mainly expressed by CNS microglia but is also expressed by monocytes, dendritic cells and natural killer cells (The Jackson Laboratory). The lack of microglia and monocytes in the CNS at the moment of SCI allowed us to investigate the origin of DAM as well as their contribution to function. Hence, a lack of DAM following SCI in Cx3cr1-depleted mice would suggest that DAM are indeed reprogrammed bMicroglia. However, if DAM would appear this would suggest that DAM are derived from peripheral monocytes entering the CNS following SCI (Fig. 9*A*). As determined by flow cytometry and immunohistochemistry, depletion was almost complete, with the percentage of CD11b^+^CX3CR1^+^ or CD11b^+^CD45^low^ at a nadir (∼0.5% of cells) 7 d after tamoxifen administration (Fig. 10*A–C*). We isolated and sequenced 604 CD45^+^ immune cells at 7 and 21 d post-SCI (14 and 28 d after full depletion, respectively). In the microglia-depleted mice, we observed a total absence of the previously observed bMicroglia-derived cells, including DAM (Fig. 9*B*,*C*). We instead observed a large increase in monocytes and monocyte-derived macrophages (mdMacrophages) expressing *Ms4a7* (Fig. 9*B–E*), instead of genes characteristic for DAM in SCI (Fig. 9*F*). Thus, when no bMicroglia were present in the spinal cord at the moment of injury DAM did not appear, in line with bMicroglia being the cell of origin for DAM.

To determine whether microglia-derived cells had an effect on recovery from injury, we concomitantly measured hindlimb locomotor function during 90 d post-SCI using the BMS (Fig. 9*A*). Over time the BMS score was significantly lower (*p* < 0.05, mixed ANOVA combined with Wilcoxon *post hoc* test) among animals subjected to depletion of Cx3cr1^+^ cells before SCI (1.83, CI: 1.51–2.16) as compared with wild-type mice (3.25, CI: 2.60–3.90) also subjected to SCI (*p* < 0.05; Fig. 9*G*). Cx3cr1^+^ cell-depleted animals which were not subjected to SCI did not deteriorate in terms of BMS score and remained at a full healthy score (9) over time (data not included). The majority of the difference in BMS score between depleted and wild-type mice was observed during the first week post-SCI, concurrent to DAM transformation. Taken together, our microglia depletion experiment shows that DAM in SCI are formed through transcriptional reprogramming of bMicroglia and have a strong beneficial effect on the recovery of hindlimb locomotor function post-SCI.

## Discussion

The present study provides a detailed description of the temporal dynamics of immune cells at the injury epicenter of traumatic SCI, at single-cell resolution. We find that following trauma to the CNS, baseline microglia undergo a distinct temporal transformation through cellular reprogramming to assume a disease-associated phenotype similar to cell states found in neurodegeneration, demyelination and development. This subtype persists during the chronic phase of disease in both rodents and humans, and contributes to functional recovery.

Baseline microglia (bMicroglia), found in healthy animals, transformed into DAM in two distinct stages with different kinetics, first transitioning within hours to an intermediary activated form (aMicroglia) and reaching a DAM-like state beginning 3 d post-SCI. The DAM state appears permanent since bMicroglia did not reappear within the 90-d period, indicating that they have undergone stable cellular reprogramming. Such stable reprogramming has previously been reported as a disease-associated transformation of microglia in both chronic and acute neurodegenerative disease conditions such as AD, demyelination, or inflammation/cell death (Keren-Shaul et al., 2017; Krasemann et al., 2017; Mathys et al., 2017; Sousa et al., 2018; Tay et al., 2018; Jordão et al., 2019; Masuda et al., 2019). In addition, a similar DAM-like population in microglia also exist in healthy developing mouse brain (Li et al., 2019). This suggests a canonical microglial activation pathway with cell death, by controlled apoptosis or injury, as the underlying trigger rather than disease-specific processes.

The origin of DAM, which appear following pathology and/or injury to the CNS, is not known. DAM could either be reprogrammed bMicroglia or the progeny of peripheral immune cells entering the CNS through the blood brain/spinal cord barrier. Our results clearly indicate that DAM are permanently reprogrammed bMicroglia, the bMicroglia cells undergo a rapid and gradual transformation into DAM, consistent with cell state transition, and depletion of bMicroglia completely abolished DAM formation. In addition, the rapid transformation of bMicroglia into aMicroglia are in line with transcriptional reprogramming rather than the slower process of proliferation of infiltrating cells. These findings are in line with Plemel et al. (2020) who found that microglia, rather than infiltrating macrophages, dominate the spinal cord following demyelination. They also found that absence of microglia during demyelination resulted in a significantly higher infiltration of peripheral macrophages. Charting the molecular mechanisms of microglial reprogramming may reveal ways of modulating their function for therapeutic purposes, promising to aid recovery in traumatic or neurodegenerative disease progression.

Our microglia depletion experiment shows that microglial activation has a strong positive effect on recovery from SCI. This is in line with previous studies which have reported that pharmacological depletion of microglia (using a CSF1R inhibitor) following SCI results in impaired functional recovery (Bellver-Landete et al., 2019). However, the fully formed DAM might not be the main effector of recovery. Whereas microglia rapidly transform during the first 2 h, they halt in an intermediate state at 6–36 h (Fig. 7*B*). During this time the cells express low levels of baseline microglial markers and slowly upregulate expression of genes associated with DAM. Considering that the majority of the functional recovery is observed already at 7 d post-SCI (i.e., before full maturation of DAM), it is plausible that this intermediate population mediates the majority of the beneficial effects resulting in enhanced functional recovery. We hypothesize that DAM (and/or intermediate populations) contribute to functional recovery by beneficially modulating the immune response following SCI. Removal of debris and dead cells as well as an appropriate inflammatory response modifying immune cell and astrocyte response is essential to minimize the glial scar and thereby promote axonal regeneration. Previous studies have mainly investigated neurodegenerative disease models (Multiple sclerosis, AD) in rodents, in which functional recovery (e.g., sensation, autonomic functions, memory) is more difficult to evaluate (Keren-Shaul et al., 2017; Krasemann et al., 2017; Mathys et al., 2017; Friedman et al., 2018; Mrdjen et al., 2018; Olah et al., 2018; Sousa et al., 2018; Tay et al., 2018; Hammond et al., 2019; Jordão et al., 2019; Masuda et al., 2019). The traumatic SCI model allows for exact and detailed evaluation of motor function, which has not previously been evaluated in a context of SCI/neurotrauma. The results presented in this study are consistent with, that DAM being beneficial for functional recovery. However, since Cx3cr1 is expressed by other immune cells in addition to microglia, we cannot exclude the possibility that for example dendritic cells or natural killer cells are the main factor beneficial for motor recovery. Irrefutable proof of the role of DAM in recovery will have to await the development of a fully specific depletion model.

Taken together, we have identified a disease-associated microglial cell type which originates from permanently reprogrammed baseline microglia via an activated cell state. These DAM cells appear within days following traumatic injury, and persist years later in human spinal cord at the site of injury. These cells seem to contribute to healing, and show a striking similarity to DAM found in neuronal degeneration or demyelination, as well as a specific microglial cell type found during development.

## References

[B1] Ajami B, Bennett JL, Krieger C, Tetzlaff W, Rossi FM (2007) Local self-renewal can sustain CNS microglia maintenance and function throughout adult life. Nat Neurosci 10:1538–1543. 10.1038/nn2014 18026097

[B2] Anders S, Pyl PT, Huber W (2014) HTSeq–a Python framework to work with high-throughput sequencing data. Bioinformatics 15 31:166–169. 10.1093/bioinformatics/btu638 PMC4287950 25260700

[B3] Basso DM, Fisher LC, Anderson AJ, Jakeman LB, McTigue DM, Popovich PG (2006) Basso mouse scale for locomotion detects differences in recovery after spinal cord injury in five common mouse strains. J Neurotrauma 23:635–659. 10.1089/neu.2006.23.635 16689667

[B4] Bellver-Landete V, Bretheau F, Mailhot B, Vallières N, Lessard M, Janelle ME, Vernoux N, Tremblay MÈ, Fuehrmann T, Shoichet MS, Lacroix S (2019) Microglia are an essential component of the neuroprotective scar that forms after spinal cord injury. Nat Commun 10:518. 10.1038/s41467-019-08446-0 30705270 PMC6355913

[B5] Butler A, Hoffman P, Smibert P, Papalexi E, Satija R (2018) Integrating single-cell transcriptomic data across different conditions, technologies, and species. Nat Biotechnol 36:411–420. 10.1038/nbt.4096 29608179 PMC6700744

[B6] Colton C, Wilcock DM (2010) Assessing activation states in microglia. CNS Neurol Disord Drug Targets 9:174–191. 10.2174/187152710791012053 20205642

[B7] Davoust N, Vuaillat C, Androdias G, Nataf S (2008) From bone marrow to microglia: barriers and avenues. Trends Immunol 29:227–234. 10.1016/j.it.2008.01.010 18396103

[B8] Dobin A, Davis CA, Schlesinger F, Drenkow J, Zaleski C, Jha S, Batut P, Chaisson M, Gingeras TR (2013) STAR: ultrafast universal RNA-seq aligner. Bioinformatics 29:15–21. 10.1093/bioinformatics/bts635 23104886 PMC3530905

[B9] Eder C, Klee R, Heinemann U (1998) Involvement of stretch-activated Cl- channels in ramification of murine microglia. J Neurosci 18:7127–7137. 9736636 10.1523/JNEUROSCI.18-18-07127.1998 PMC6793253

[B10] Friedman BA, Srinivasan K, Ayalon G, Meilandt WJ, Lin H, Huntley MA, Cao Y, Lee S-H, Haddick PCG, Ngu H, Modrusan Z, Larson JL, Kaminker JS, van der Brug MP, Hansen DV (2018) Diverse brain myeloid expression profiles reveal distinct microglial activation states and aspects of Alzheimer's disease not evident in mouse models. Cell Rep 22:832–847. 10.1016/j.celrep.2017.12.066 29346778

[B11] Hammond TR, Dufort C, Dissing-Olesen L, Giera S, Young A, Wysoker A, Walker AJ, Gergits F, Segel M, Nemesh J, Marsh SE, Saunders A, Macosko E, Ginhoux F, Chen J, Franklin RJM, Piao X, McCarroll SA, Stevens B (2019) Single-cell RNA sequencing of microglia throughout the mouse lifespan and in the injured brain reveals complex cell-state changes. Immunity 50:253–271.e6. 10.1016/j.immuni.2018.11.004 30471926 PMC6655561

[B12] Hanisch UK, Kettenmann H (2007) Microglia: active sensor and versatile effector cells in the normal and pathologic brain. Nat Neurosci 10:1387–1394. 10.1038/nn1997 17965659

[B13] Jordão MJC, Sankowski R, Brendecke SM, Sagar, Locatelli G, Tai YH, Tay TL, Schramm E, Armbruster S, Hagemeyer N, Groß O, Mai D, Çiçek Ö, Falk T, Kerschensteiner M, Grün D, Prinz M (2019) Single-cell profiling identifies myeloid cell subsets with distinct fates during neuroinflammation. Science 363:eaat7554. 10.1126/science.aat7554 30679343

[B14] Keren-Shaul H, Spinrad A, Weiner A, Matcovitch-Natan O, Dvir-Szternfeld R, Ulland TK, David E, Baruch K, Lara-Astaiso D, Toth B, Itzkovitz S, Colonna M, Schwartz M, Amit I (2017) A unique microglia type associated with restricting development of Alzheimer's disease. Cell 169:1276–1290.e17. 10.1016/j.cell.2017.05.018 28602351

[B15] Kettenmann H, Hanisch UK, Noda M, Verkhratsky A (2011) Physiology of microglia. Physiol Rev 91:461–553. 10.1152/physrev.00011.2010 21527731

[B16] Krasemann S, Madore C, Cialic R, Baufeld C, Calcagno N, El Fatimy R, Beckers L, O'Loughlin E, Xu Y, Fanek Z, Greco DJ, Smith ST, Tweet G, Humulock Z, Zrzavy T, Conde-Sanroman P, Gacias M, Weng Z, Chen H, Tjon E, et al. (2017) The TREM2-APOE pathway drives the transcriptional phenotype of dysfunctional microglia in neurodegenerative diseases. Immunity 47:566–581.e9. 10.1016/j.immuni.2017.08.008 28930663 PMC5719893

[B17] Li Q, Cheng Z, Zhou L, Darmanis S, Neff NF, Okamoto J, Gulati G, Bennett ML, Sun LO, Clarke LE, Marschallinger J, Yu G, Quake SR, Wyss-Coray T, Barres BA (2019) Developmental heterogeneity of microglia and brain myeloid cells revealed by deep single-cell RNA sequencing. Neuron 101:207–223.e10. 10.1016/j.neuron.2018.12.006 30606613 PMC6336504

[B18] Liao Y, Smyth GK, Shi W (2014) featureCounts: an efficient general-purposeprogram for assigning sequence reads to genomic features. Bioinformatics 30:923–930. 24227677 10.1093/bioinformatics/btt656

[B19] Masuda T, Sankowski R, Staszewski O, Böttcher C, Amann L, Sagar, Scheiwe C, Nessler S, Kunz P, van Loo G, Coenen VA, Reinacher PC, Michel A, Sure U, Gold R, Grün D, Priller J, Stadelmann C, Prinz M (2019) Spatial and temporal heterogeneity of mouse and human microglia at single-cell resolution. Nature 566:388–392. 10.1038/s41586-019-0924-x 30760929

[B20] Mathys H, Adaikkan C, Gao F, Young JZ, Manet E, Hemberg M, De Jager PL, Ransohoff RM, Regev A, Tsai LH (2017) Temporal tracking of microglia activation in neurodegeneration at single-cell resolution. Cell Rep 21:366–380. 10.1016/j.celrep.2017.09.039 29020624 PMC5642107

[B21] Mildner A, Schmidt H, Nitsche M, Merkler D, Hanisch UK, Mack M, Heikenwalder M, Brück W, Priller J, Prinz M (2007) Microglia in the adult brain arise from Ly-6ChiCCR2+ monocytes only under defined host conditions. Nat Neurosci 10:1544–1553. 10.1038/nn2015 18026096

[B22] Mrdjen D, Pavlovic A, Hartmann FJ, Schreiner B, Utz SG, Leung BP, Lelios I, Heppner FL, Kipnis J, Merkler D, Greter M, Becher B (2018) High-dimensional single-cell mapping of central nervous system immune cells reveals distinct myeloid subsets in health, aging, and disease. Immunity 48:380–395. e386. 10.1016/j.immuni.2018.01.011 29426702

[B23] Olah M, Patrick E, Villani AC, Xu J, White CC, Ryan KJ, Piehowski P, Kapasi A, Nejad P, Cimpean M, Connor S, Yung CJ, Frangieh M, McHenry A, Elyaman W, Petyuk V, Schneider JA, Bennett DA, De Jager PL, Bradshaw EM (2018) A transcriptomic atlas of aged human microglia. Nat Commun 9:539. 10.1038/s41467-018-02926-5 29416036 PMC5803269

[B24] O'Koren EG, Yu C, Klingeborn M, Wong AYW, Prigge CL, Mathew R, Kalnitsky J, Msallam RA, Silvin A, Kay JN, Bowes Rickman C, Arshavsky VY, Ginhoux F, Merad M, Saban DR (2019) Microglial function is distinct in different anatomical locations during retinal homeostasis and degeneration. Immunity 50:723–737.e7. 10.1016/j.immuni.2019.02.007 30850344 PMC6592635

[B25] Picelli S, Faridani OR, Björklund AK, Winberg G, Sagasser S, Sandberg R (2014) Full-length RNA-seq from single cells using Smart-seq2. Nat Protoc 9:171–181. 10.1038/nprot.2014.006 24385147

[B26] Plemel JR, Stratton JA, Michaels NJ, Rawji KS, Zhang E, Sinha S, Baaklini CS, Dong Y, Ho M, Thorburn K, Friedman TN, Jawad S, Silva C, Caprariello AV, Hoghooghi V, Yue J, Jaffer A, Lee K, Kerr BJ, Midha R, et al. (2020) Microglia response following acute demyelination is heterogeneous and limits infiltrating macrophage dispersion. Sci Adv 6:eaay6324. 10.1126/sciadv.aay6324 31998844 PMC6962036

[B27] Ritchie ME, Phipson B, Wu D, Hu Y, Law CW, Shi W, Smyth GK (2015) limma powers differential expression analyses for RNA-sequencing and microarray studies. Nucleic Acids Res 43:e47. 10.1093/nar/gkv007 25605792 PMC4402510

[B28] Robinson MD, McCarthy DJ, Smyth GK (2010) edgeR: a bioconductor package for differential expression analysis of digital gene expression data. Bioinformatics 26:139–140. 10.1093/bioinformatics/btp616 19910308 PMC2796818

[B29] Schilling T, Nitsch R, Heinemann U, Haas D, Eder C (2001) Astrocyte-released cytokines induce ramification and outward K+ channel expression in microglia via distinct signalling pathways. Eur J Neurosci 14:463–473. 10.1046/j.0953-816x.2001.01661.x 11553296

[B30] Sievers J, Parwaresch R, Wottge HU (1994) Blood monocytes and spleen macrophages differentiate into microglia-like cells on monolayers of astrocytes: morphology. Glia 12:245–258. 10.1002/glia.440120402 7890329

[B31] Sousa C, Golebiewska A, Poovathingal SK, Kaoma T, Pires-Afonso Y, Martina S, Coowar D, Azuaje F, Skupin A, Balling R (2018) Single-cell transcriptomics reveals distinct inflammation-induced microglia signatures. EMBO Rep 19:e46171. 10.15252/embr.201846171 30206190 PMC6216255

[B32] Tay TL, Sagar, Dautzenberg J, Grün D, Prinz M (2018) Unique microglia recovery population revealed by single-cell RNAseq following neurodegeneration. Acta Neuropathol Commun 6:87. 10.1186/s40478-018-0584-3 30185219 PMC6123921

[B33] Team BC (2015) Mus.musculus: annotation package for the Mus musculus object. https://bioconductor.org/packages/release/data/annotation/html/Mus.musculus.html.

[B34] Team RC (2016) R: a language and environment for statistical computing. R Foundation for Statistical Computing.

[B35] Wang Y, Cella M, Mallinson K, Ulrich JD, Young KL, Robinette ML, Gilfillan S, Krishnan GM, Sudhakar S, Zinselmeyer BH, Holtzman DM, Cirrito JR, Colonna M (2015) TREM2 lipid sensing sustains the microglial response in an Alzheimer's disease model. Cell 160:1061–1071. 10.1016/j.cell.2015.01.049 25728668 PMC4477963

