# Supplementary figures and images for: Spinal Cord Injury Induces Permanent Reprogramming of Microglia into a Disease-Associated State Which Contributes to Functional Recovery
Source: J Neurosci. 2021 Oct 6;41(40):8441–59. doi: 10.1523/JNEUROSCI.0860-21.2021 (PMC8496189; doi:10.1523/JNEUROSCI.0860-21.2021)

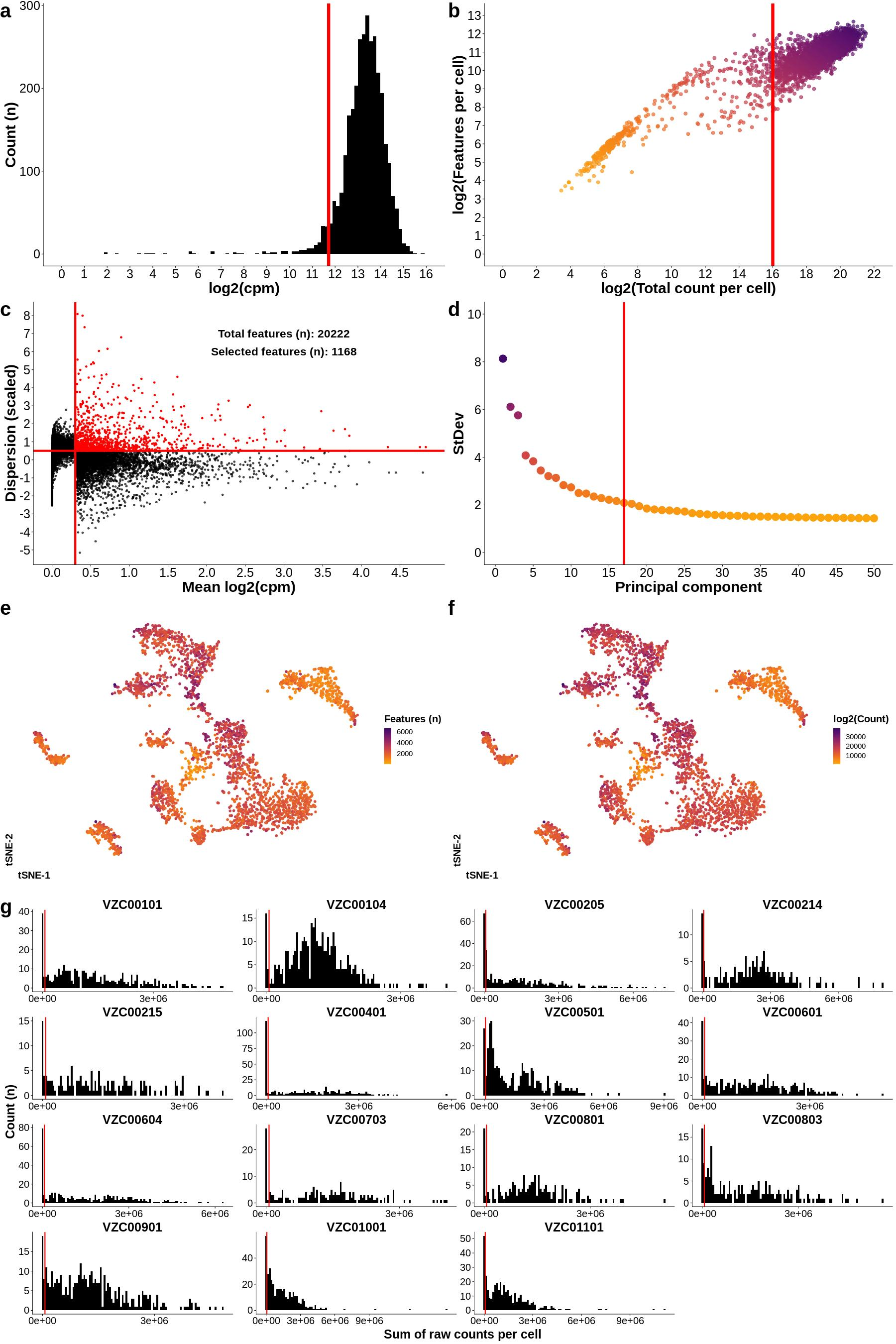

Supplement: Extended Data Figure 1-1 — A, Histogram (100 bins) of log2(cpm) of Actb expression in each cell. Red line indicates the 1st percentile. All cells with Actb expression > 1st percentile threshold were included in the analysis. B, log2-log2 plot of number of features and total number of counts per cell. Red line indicates a minimum total count of 216 per cell. All cells with a total count exceeding 216 were included in the analysis. C, Scaled dispersion for each gene plotted against the mean log2(cpm) expression for each gene. A total 1168 genes indicated with red color were selected as highly variable genes and used in the downstream analysis pipeline. D, Standard deviation for the first 50 PCs. Red line indicates the 17 first components, which were deemed to be the most variable components and therefore included in the FindNeighbors() function when clustering cells. E, tSNE plot of 3069 CD45+ immune cells which passed the quality control. Number of features per cell is indicated using a continuous color. F, Equivalent to E and indicates the number of genes with a positive value for log2(count) per cell using a continuous color. G, Histograms of the total number of counts per cell for each 384-well plate included in the analysis. Red line indicates the 216 threshold determined in B. Download Figure 1-1, TIF file. [file ns-JN-RM-0860-21-s01.tif]

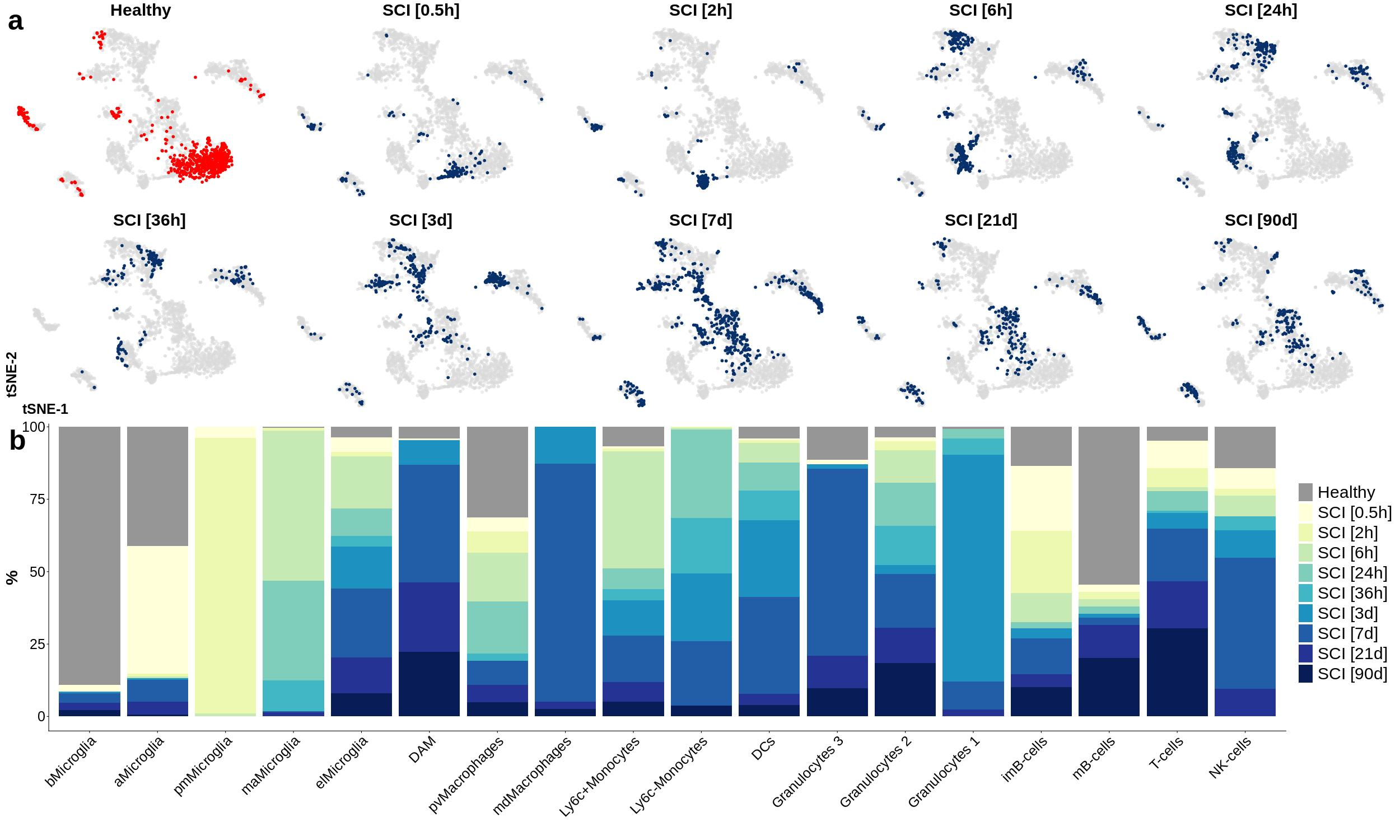

Supplement: Extended Data Figure 1-2 — A, tSNE plots of 3069 CD45+ immune cells in SCI. Each plot corresponds to a time point post-SCI. Healthy tissue is used as reference. B, Proportion of cells within each cell type associated with a specific time point of evaluation. Healthy tissue is used as reference. Download Figure 1-2, TIF file. [file ns-JN-RM-0860-21-s02.tif]

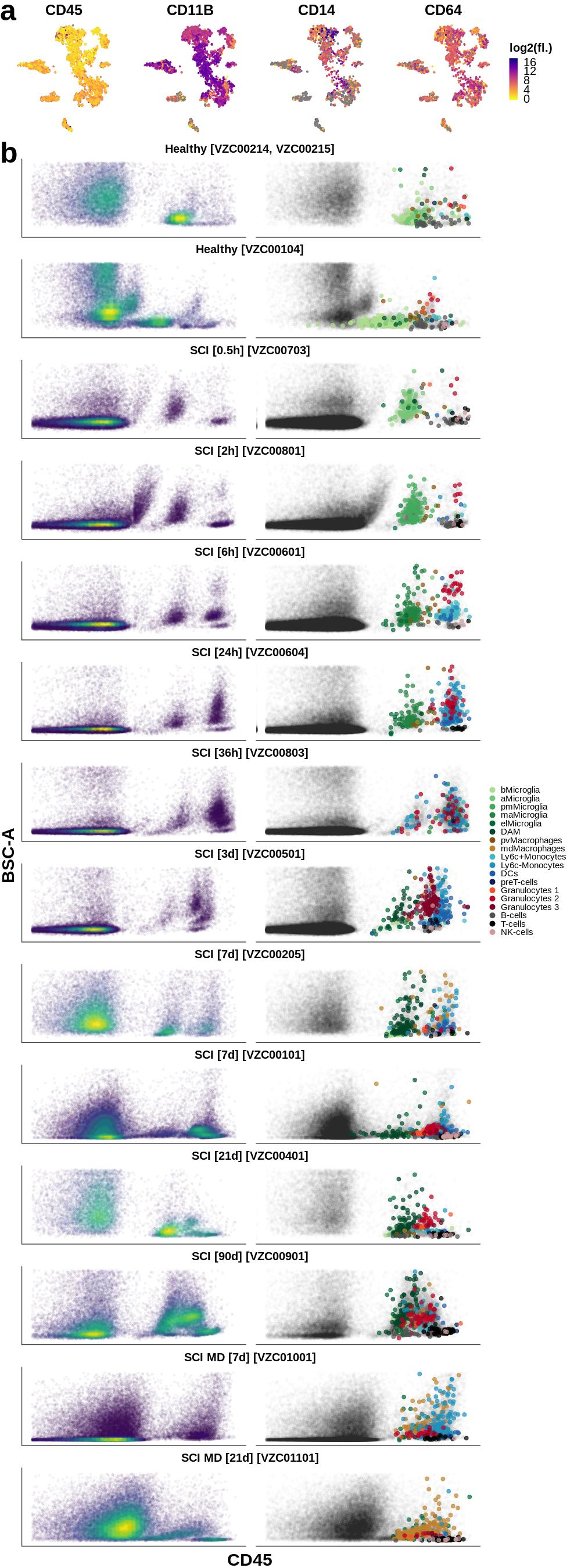

Supplement: Extended Data Figure 1-3 — A, tSNE plots of 3069 CD45+ immune cells following index sorting using surface markers CD11b, CD45, CD14, and CD64. Log2(fluorescence) is represented with a continuous color. B, FACS plots of CD45 surface expression versus BSC-A for each 384-well plate separately (time point post-SCI indicated in plot). Leftmost FACS plots are density plots reporting all analyzed cells. Rightmost plots are equivalent to leftmost plots with sorted and sequenced cells annotated based on cell type using color. bMicroglia: baseline microglia; aMicroglia: activated microglia; pmMicroglia: proliferation-mediating microglia; maMicroglia: monocyte-activating microglia; DAM: disease-associated microglia in SCI; elMicroglia: embryonic-like microglia; pvMacrophages: perivascular macrophages; mdMacrophages: monocyte-derived macrophages; DCs: dendritic cells; imB-cells: immature B-cells; mB-cells: mature B-cells. Download Figure 1-3, TIF file. [file ns-JN-RM-0860-21-s03.tif]

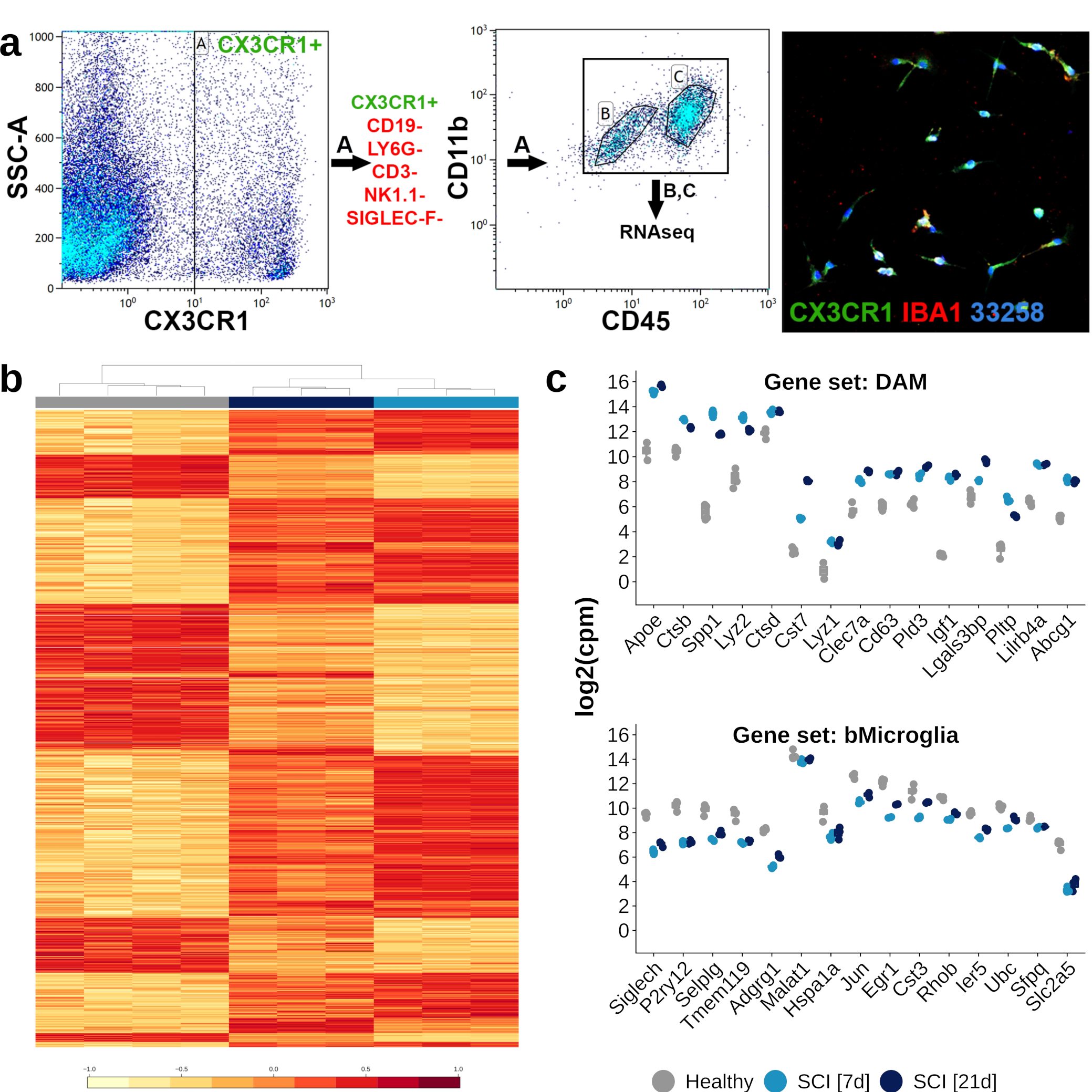

Supplement: Extended Data Figure 1-4 — A, CX3CR1+CD19–Ly6G–CD3–NK1.1–SIGLECF–CD11b+CD45high/low cells were sorted from SCI from which RNA was sequenced in bulk at 7 d (n = 3) and 21 d (n = 3) post-SCI. Healthy tissue was used as reference (n = 4). Sorted cells were plated and cultured for 72 h and stained for IBA1. B, Agglomerative hierarchical clustering with heat map representation of biological replicates for 3323 significantly differentially expressed genes (FDR < 0.01; logFC<-1 OR logFC > 1) for all three contrasts. Condition indicated with color and reported in C. C, log2(cpm) expression for the top 15 upregulated and downregulated genes in DAM in SCI and baseline microglia (bMicroglia). Download Figure 1-4, TIF file. [file ns-JN-RM-0860-21-s04.tif]

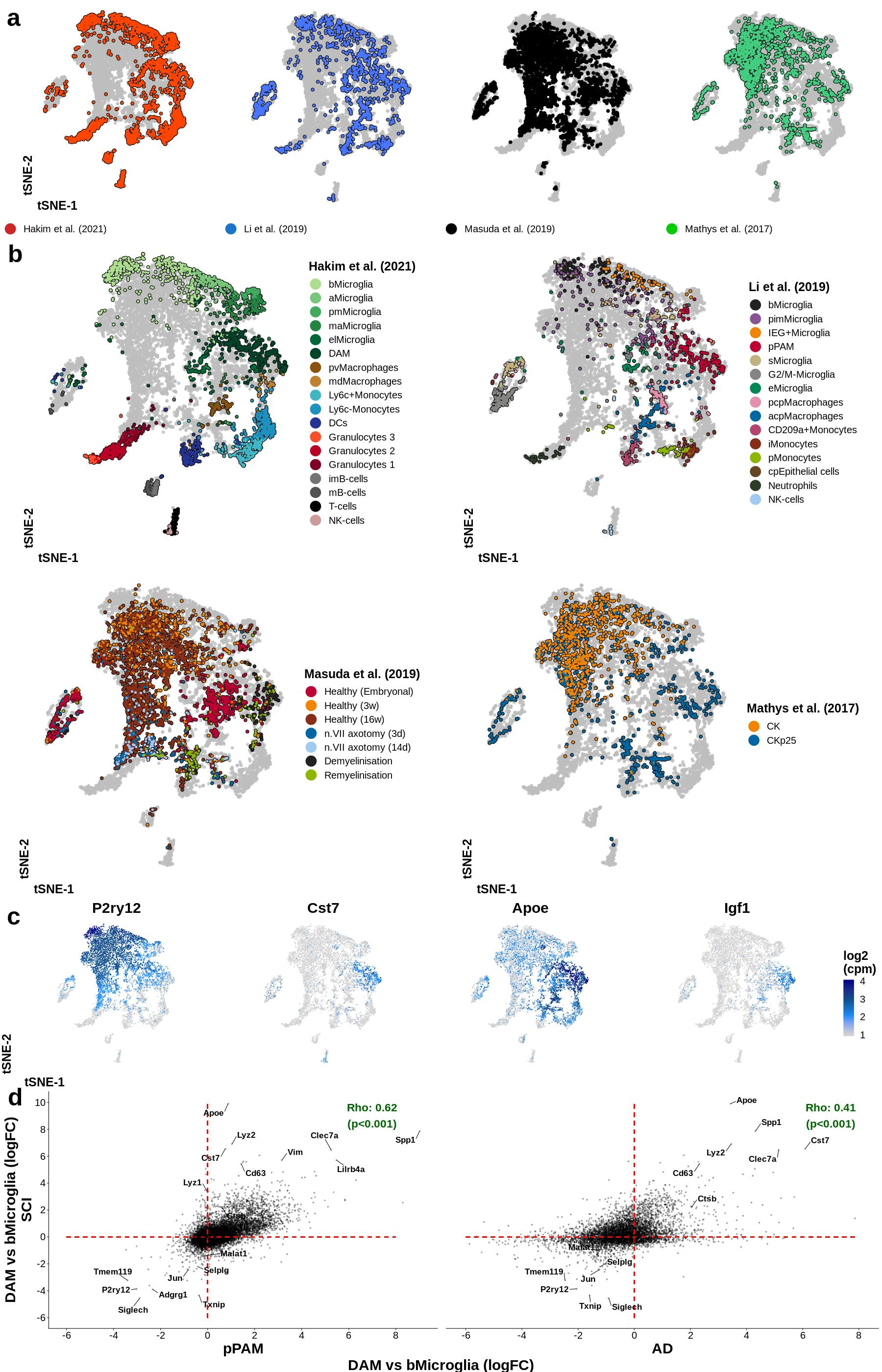

Supplement: Extended Data Figure 8-1 — A, tSNE plot of 10014 immune and microglial cells following integration of the data in this study with three published datasets. B, Cells from one study annotated in each tSNE using the original metadata. bMicroglia: baseline microglia; aMicroglia: activated microglia; pmMicroglia: proliferation-mediating microglia; maMicroglia: monocyte-activating microglia; DAM: disease-associated microglia in SCI; elMicroglia: embryonic-like microglia; pvMacrophages: perivascular macrophages; mdMacrophages: monocyte-derived macrophages; DCs: dendritic cells; imB-cells: immature B-cells; mB-cells: mature B-cells; pimMicroglia: postnatal immature microglia; IEG+Microglia: immediate early genes microglia; sMicroglia: s-phase microglia; pPAM: postnatal proliferative-region-associated microglia; eMicroglia: embryonic microglia; pcpMacrophages: postnatal choroid plexus macrophages; acpMacrophages: adult choroid plexus macrophages; iMonocytes: inflammatory monocytes; pMonocytes: patrolling monocytes; cpEpithelial cells: choroid plexus epithelial cells. C, tSNE plots of 10014 immune and microglial cells reporting log2(cpm) expression of selected genes with a continuous color for each cell separately. D, logFC for contrast DAM versus bMicroglia correlated between SCI, pPAM, and AD. Download Figure 8-1, TIF file. [file ns-JN-RM-0860-21-s05.tif]
